# Multiple stressor effects on marine infauna: responses of estuarine taxa and functional traits to sedimentation, nutrient and metal loading

**DOI:** 10.1038/s41598-017-12323-5

**Published:** 2017-09-20

**Authors:** J. I. Ellis, D. Clark, J. Atalah, W. Jiang, C. Taiapa, M. Patterson, J. Sinner, J. Hewitt

**Affiliations:** 1 Cawthron Institute, Private Bag 2, Nelson, 7042 New Zealand; 2 King Abdullah University of Science and Technology (KAUST), Red Sea Research Center, Division of Biological and Environmental Science and Engineering, Thuwal, 23955-6900 Saudi Arabia; 3 Manaaki Te Awanui, 234a Waihi Rd, Tauranga, 3110 New Zealand; 4 Massey University, School of People Environment and Planning, Private Bag 11222, Palmerston North, 4442 New Zealand; 5 National Institute of Water and Atmospheric Research, PO Box 11115, Hillcrest, Hamilton, 3216 New Zealand

## Abstract

Sedimentation, nutrients and metal loading to coastal environments are increasing, associated with urbanization and global warming, hence there is a growing need to predict ecological responses to such change. Using a regression technique we predicted how maximum abundance of 20 macrobenthic taxa and 22 functional traits separately and interactively responded to these key stressors. The abundance of most taxa declined in response to sedimentation and metal loading while a unimodal response was often associated with nutrient loading. Optimum abundances for both taxa and traits occurred at relatively low stressor levels, highlighting the vulnerability of estuaries to increasing stressor loads. Individual taxa were more susceptible to stress than traits, suggesting that functional traits may be less sensitive for detecting changes in ecosystem health. Multiplicative effects were more common than additive interactions. The observed sensitivity of most taxa to increasing sedimentation and metal loading and the documented interaction effects between multiple stressors have important implications for understanding and managing the ecological consequences of eutrophication, sedimentation and contaminants on coastal ecosystems.

## Introduction

Elevated sedimentation, nutrients and metal loading to estuarine and coastal environments can occur via urban development, forestry, farming and inadequate land management practices. These stressors can cause broad-scale changes in estuarine and coastal ecology by modifying habitats and influencing the health, abundance and distribution of functionally important species. Synergistic effects are likely to occur as multiple stressors impact coastal ecosystems^1,2^ and these interactions are predicted to increase as climate change influences the delivery of freshwater and associated sediments, nutrients and metals to the coastal zone^3–6^. Understanding the response of benthic communities to key stressors is important for managing our coastal environments and the first step towards setting ecologically relevant limits for sedimentation, nutrients and metal loading.

Human activities in coastal watersheds provide the major sources of sediment, nutrients and metals entering shallow coastal ecosystems. Increases in sediment loads have been recognised as a marine contaminant of considerable importance^7^ and can occur as a result of urban development, forestry, farming and inadequate land management practices^8,9^. Elevated sediment loading to coastal environments has been found to lead to broad scale changes in the ecology from habitat modification^10,11^ through to functional changes including a loss of key suspension feeding species and a switch to deposit feeding communities^9,12^.

Increased delivery of nutrients to coastal waters can occur, for example, due to leaching of fertilizer and manure into waterways from agricultural land, discharges from waste water treatment facilities and deforestation^13^. Low levels of nutrient enrichment in estuarine and coastal environments can have a positive effect on the benthos due to improved primary productivity, and therefore food availability. Beyond a critical point, however, excessive nutrient discharges can lead to accelerated eutrophication of coastal environments and adverse symptoms of over enrichment^14,15^.

The major terrestrial sources of anthropogenic metals to coastal areas are municipal and industrial discharges, mining and urban development. Urban stormwater in particular has been found to be a significant contemporary source of heavy metals^16^. Metals can be essential for organisms as trace elements, however at higher concentrations they can become toxic^17^. High exposure to heavy metals can cause physiological stress, reduced reproductive success, and outright mortality in associated invertebrates and fishes^18–22^. Estuaries and coastal ecosystems are particularly vulnerable to these stressors as they act as natural retention systems for sediments and heavy metal contaminants and are affected by nutrient run-off^23^.

Communities shift under different stressor loads, thus by following communities we may be able to understand relationships between communities and stressor levels and predict the changes to ecosystem health and resilience. In ecology, it is common for data points to be scattered beneath an upper (or above a lower) limit described as a ‘factor ceiling’^24^. The ceiling to the data scatter implies a constraining factor, thus the form the ceiling takes allows us to derive maximum (or minimum) possible response curves to an environmental variable. This implies that over broad scales, while a number of factors (*e.g*. the potential for recruitment, historical conditions etc.) may affect the observed density, there is a limit to abundance (frequently an upper limit) that is controlled by the variable of interest.

Correlative approaches that use environmental information to explain and predict patterns of species distribution are broadly referred to as species distribution models^25^. These predictive modeling methods have been primarily used in terrestrial ecology to study general patterns of species distribution, with the use of species distribution models in marine ecosystems still in its infancy^26^. Actual and potential management applications of species distribution models include testing hypotheses in relation to the ranges of species distribution along environmental gradients, creating habitat suitability maps that predict the specific ecological potential of a habitat and/or identify ecologically important areas in need of protection^27^, assessing the possible consequences of habitat change including climate change scenario modeling^28^, designing cost-effective monitoring programs^29^ and quantitatively assigning ecological groupings for biotic health indices (e.g. AZTI Marine Biotic Index, AMBI^30^).

While species distribution models are critical for sustainable management, another complementary approach is to assess functional changes or functional traits based approaches. With the recent shift toward more holistic marine ecosystem management approaches there has been increasing attention on assessing ecosystem function as a complement to evaluations based on species composition and abundance^31–34^. Different species may perform similar ecological roles and the functional structure of a community can be represented by a series of life history, morphological and behavourial characteristics, known as functional traits, which determine how the constituent species influence biological processes. For example, deep burrowing fauna significantly influence rates and pathways of organic matter mineralization^35^. Functional approaches allow generalisations to be made across areas with differing species compositions and provide clearer mechanistic links to ecosystem services, which may be more meaningful to managers in terms of ecosystem health. Models that link traits with environmental conditions can predict how future ecosystem function will change under different scenarios. As with species distribution modeling, functional trait assessments are a relatively new concept in marine management and have primarily focused on describing community functional diversity and redundancy^36–38^. Thus far, there are no published studies that we know of that investigate the response of individual functional traits to sedimentation, nutrient and metal loading in marine systems. Therefore, a second aim of this paper was to assess not only species-specific responses but also functional traits responses to changing stressor levels.

Finally, stressors such as sedimentation, nutrients and metal loading do not affect species distributions or functional traits in isolation. Marine communities are usually subject to multiple co-occurring human activities^39–41^. While the individual effects of single stressors on species and ecosystem function has been studied, there has been less research into the cumulative and interactive effects of multiple stressors^1,42^ which generally influence coastal marine ecosystems. Predicting multiple cumulative stressor effects is challenging given the range of possible stressors and their potential to interact in a variety of ways. Thrush *et al*.^43^ suggest the best way to understand the potential for multiple stressor effects is to analyse responses, across gradients of environmental stressors, over a large number of species. Understanding the potential for multiple stressor effects to occur, and the nature of those interactions, would improve ecological risk assessments and subsequent resource management decisions^44^.

The aim of the present study was to therefore investigate; i) how individual stressors affect macrofauna abundance, ii) how these individual stressors affect functional traits and iii) the potential for multiple stressor interactions. We developed maximum abundance models to forecast how the abundances of individual species are likely to change in response to three key stressors; sedimentation, nutrients and metal loading. In order to assess how these stressors affected ecosystem functioning, we also developed models to predict how functional traits change along these stressor gradients. Finally, multiple regression was used to investigate whether the major stressors interacted and influenced the abundance of common macrofauna and functional traits.

## Methods

### Study site

Tauranga Harbour is a large (approximately 200 km^2^), well flushed barrier enclosed lagoon located on New Zealand’s North Island^45^. The harbour is predominantly shallow (<10 m deep), with intertidal flats comprising approximately 66% of the total area. Catchment land use is predominantly pasture and indigenous forest with urbanisation concentrated in the south-east, near the city of Tauranga. Sampling was carried out from December 2011 to February 2012. A total of 75 sites across the harbour were sampled for benthic macrofauna and associated sediment characteristics.

**Figure 1:**
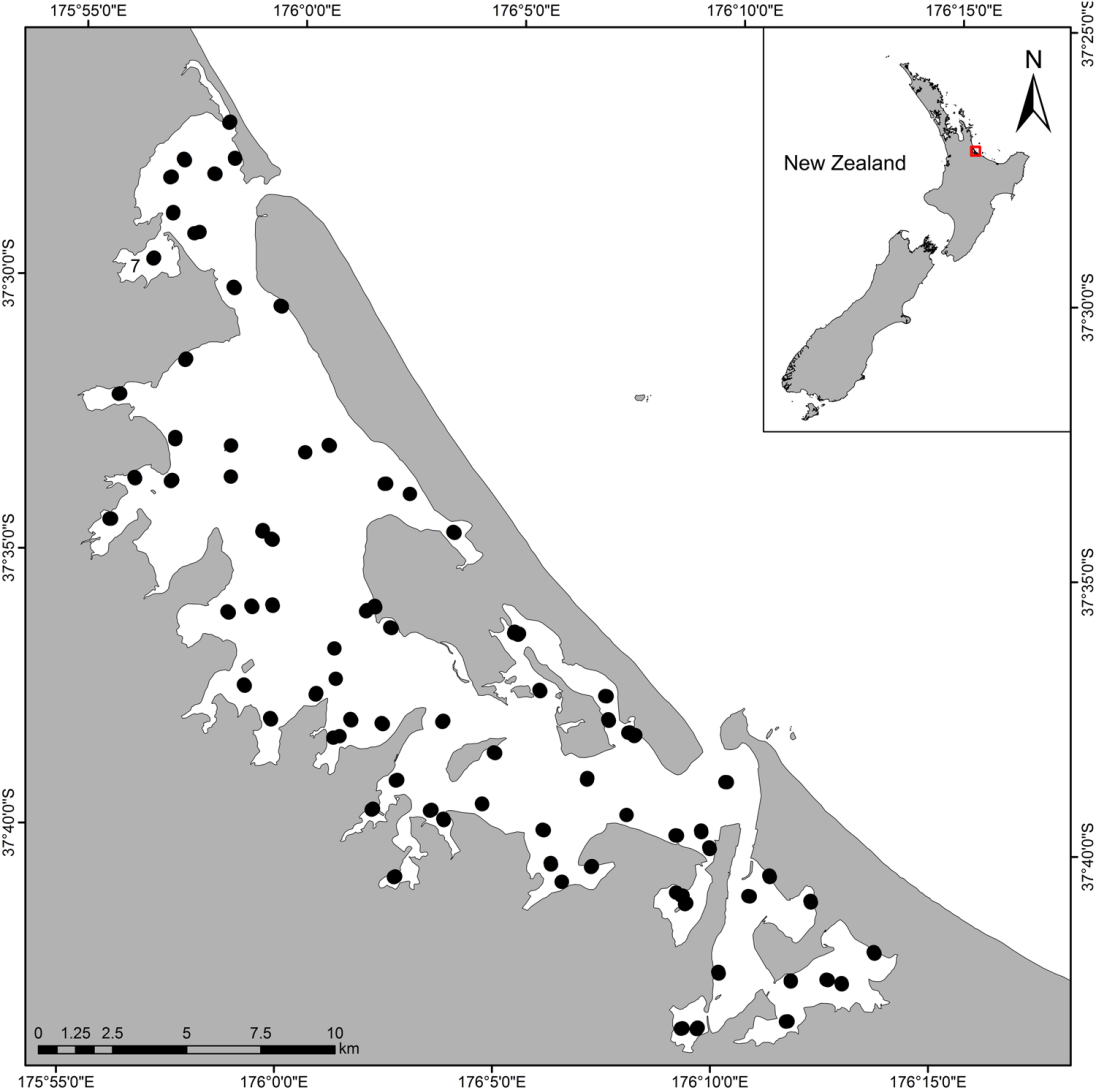
Map of Tauranga Harbour showing locations of the 75 sampling sites. This figure was created using ArcGIS software (ArcMap 10.4.1) by ESRI.

All sites were intertidal with relatively similar daily fluctuations in depth, temperature and salinity. Salinity (28–34 PSU) and temperature (18–22 °C in summer) are relatively constant across the harbour^46^. At each site, a 2 × 5 grid of ten plots (each 10 m × 10 m) was marked out and a replicate collected from each plot, yielding 750 samples overall (Fig. 1).

### Physico-chemical variables

At each site, one 2 cm diameter core extending 2 cm deep into the sediment was collected from each of the 10 plots in the grid yielding 10 replicates for each site. The replicates were composited into a single sample and the sediment was analysed for grain size, organic matter (loss on ignition, LOI), nutrients (total nitrogen, TN; total phosphorous, TP), metals (lead, Pb; zinc, Zn; copper, Cu) and chlorophyll-*α* (chl-*α*). Ellis *et al*.^23^ provides detailed methodology for the analysis of these physico-chemical variables.

### Infauna

Infauna samples were collected to quantify benthic community structure at each site. One 13 cm diameter core extending 15 cm into the sediment was taken from each of the 10 plots in the grid yielding 10 replicates for each site. Core samples were sieved on a 1 mm mesh and macrofauna retained on the sieves was preserved with ethanol then sorted and identified to the lowest practicable taxonomic resolution.

### Identifying key stressors

Preliminary analysis using multivariate linear regression to select variables that explain the maximum variation in the community data cloud was performed using Distance based Linear Modelling DistLM^47^. DistLM was analysed using square-root transformed Bray-Curtis similarities with a backward selection procedure based on the Akaike’s Information Criterion (AIC) selection criteria. Variables included in the analysis were percentage mud (particles < 63 μm), LOI, TN, TP, Pb, Cu, Zn, chl-*α*. This analysis identified percentage mud as the key grain size variable explaining benthic community composition, which was subsequently used as the stress gradient for sedimentation. For nutrients and metal loading, a number of correlated variables were determined to be important; TP and TN for nutrients and Cu and Pb for metal loading. Principal component analyses (PCA) were used to reduce the set of correlated variables to orthogonal (uncorrelated) axes^48^ for nutrients and metals separately. In both cases, the first principal component axis was used to characterise an overall gradient corresponding to increases in the concentrations of nutrients (i.e. TN and TP) and metals (i.e. Cu and Pb). PCAs were performed on the basis of square-root and log-transformed nutrient and metal concentrations, respectively. The first PCA axis (PC1) explained 94.8% and 93.5% of the total variance in the nutrients and metals data, respectively.

### Modelling taxon-specific responses to stressors

Of the 117 taxa recorded, taxa were considered for modelling if they were present at more than 10 of the 75 sites and in numbers of at least two individuals per replicate (on average). Twenty taxa were chosen providing a reasonable dataset from which to develop models and representing a range of phyla, life-histories and functional groups that are common to intertidal estuaries.

Maximum abundances across gradients of sedimentation, nutrients and metal loading were modelled using the method proposed by Blackburn *et al*.^49^. For these models, each of the three variables representing pressure gradients (percentage mud, PC1 nutrients and PC1 metals) were divided into bins and the maximum abundance of each taxon in each bin determined. There were 20 bins for sedimentation, 21 for nutrients and 30 for metal loading, with intervals of 4.0, 0.5 and 0.33 used, respectively. Each bin included no more than 20 observations, and there were roughly equal numbers of observations in at least three bins.

Generalised Linear Models (GLMs) with poisson errors and log link were then fitted to the data, using the maximum abundance in each bin as the dependent variable and the number of observations in each bin as weights. Percentage mud, PC1 nutrients and PC1 metals were included as the independent variables with up to two degree polynomials. However, the number of predictor variables and higher degree terms entered into the final model was based on the AIC selection. Data transformation (either log or square root) was applied to the independent variable if it decreased the AIC or increased *R* ^2^. The final model used for each taxon was that function which explained the most variability. All analyses were conducted using the R software package^50^.

### Modelling functional responses to stressors

The response of functional traits to increasing sedimentation, nutrients and metal loading were investigated for eight trait categories; motility, feeding, habitat structure, sediment movement, size, form, living position in sediment and body hardness with individual traits within each category (Table 1; modified from^33^) . Information for assigning taxa to functional traits was derived from taxonomic and natural history texts, body characteristics (e.g. jaw structures) and personal observations from video and core processing. While for some general categories (e.g. size and shape) taxa were only allocated to one trait (e.g. small or large), for others taxa could either exhibit multiple traits (e.g. feeding as a predator and a scavenger) or not exhibit a trait at all (e.g. sediment structuring). Individual taxa (*n* = 117) were then scored for the extent to which they displayed the categories of these traits using a ‘fuzzy coding’ procedure^51^, with allocation across each trait category summing to 1.

**Table 1:** Traits based on species behaviour, size, shape and hardness, nominated as influencing either directly or indirectly important ecosystem functions and selected for modelling (modified from^33^). Traits are listed in order of mean abundance across the dataset for each functional trait category.

Functional trait category	Trait
Motility	Freely motile in or on sediment
	Sedentary or only moving within fixed tube structures
	Limited free movement, e.g. withdrawal into sediment
Feeding	Deposit
	Suspension
Habitat structure	Simple hole or pit
	Permanent burrow
	Trough - producing troughs in sediment
	Tube
Sediment particle movement	Surface mixing
	Deep-to-surface
	Surface (top 2 cm)-to-deep (>2 cm deep)
Body size	Small (0.5–5 mm longest dimension, exclusive)
	Large (> = 20 mm longest dimension)
	Medium (5–20 mm longest dimension, exclusive)
Body form	Round/globulose (length 1–3 × width)
	Worm-shaped (length 10–100 × width)
Living position in sediment	Top 2 cm
	Deeper than 2 cm
	Attached to other animals or small hard surfaces
Body hardness	Soft-bodied
	Calcified (fully calcified shell; molluscs)

Seventy percent of taxa were matched exactly to trait information at species or genus level and 21% of taxa were not identified to less than family level so were matched exactly to trait information at higher levels of taxonomic resolution. Trait information for individual genera or species was not available for 9% of taxa so trait information from other species in the same family was used. If different species within a family had been recorded as exhibiting different traits, the taxon was assigned equal probabilities of belonging to the range exhibited by the broad taxonomic group.

The information on taxa abundance for each replicate and functional trait scores for each taxon were then combined using a weighting procedure. To do this, the abundance of each taxon was multiplied by its fuzzy coding for each trait. The category scores were then summed over all taxa present in each replicate, resulting in a replicate by trait table containing the overall frequencies of occurrence of functional traits in each replicate^52^.

Of the 32 traits recorded, traits were considered for modelling if they were present at more than 10 of the 75 sites and in numbers of more than four abundance-weighted traits per replicate (on average). Twenty-two traits met these criteria providing a reasonable dataset from which to develop models that represented a wide range of functional forms.

Maximum abundances of functional traits across gradients of sedimentation, nutrients and metal loading were modelled in the same way as the taxon-specific responses (Section 2.5).

### Calculating the distribution and optimum ranges

Distribution and optimum ranges were calculated for each modelled taxon and functional trait as a function of the sedimentation, nutrient and metal loading gradients. The distribution range was defined as the stressor (percentage mud, PC1 nutrients, PC1 metals) range over which at least one individual occurred, and was obtained using raw abundance data. The optimum was defined as the stressor ranges over which taxa or traits exhibit their maximum density and was estimated using the density maxima models. Following the methods of Robertson *et al*.^30^, a cut-off point was set at the upper 40% of the raw (non-zero) abundance data, because this value provided a balance between stressor values with low abundance (i.e. suboptimal conditions) and stressor values with high abundance (i.e. highly preferred conditions). To convert the PC1 nutrient and PC1 metals to individual variable values (i.e. TN, TP, Cu, Pb), the PC1 values were regressed against the variable of interest and the resulting equations used to work out the concentration of a given variable.

### Multiple stressors

We were interested in the potential for multiple stressor effects to occur due to the interaction of sedimentation, nutrients and metal loading. We therefore used multiple regression models to determine the relationships between the abundances of individual taxa or functional traits and sediment mud content, nutrients and metal concentrations, identifying multiple stressor effects through the significance of appropriate interaction terms. These models were only intended to be used to explore the prevalence and nature of stressor interactions, not to make predictions about taxa and trait abundances.

Infauna data was used at the replicate level (750 replicates) and initial models included all variables and their pairwise interaction terms. Models also included two degree polynomial terms to account for any unimodal responses. Predictor variables used in the multiple regression models included three stressors: percentage mud (sedimentation), PC1 nutrients (nutrients) and PC1 metals (metal loading). Count data of taxa abundance was generally over-dispersed and had a large proportion of zero data, thus models were fitted using negative binomial errors with a log link function. Functional traits data was strictly positive continuous data, thus it was modelled using Gamma errors with a log link function. Final models (i.e. the most parsimonious) for both taxa and traits were backwards selected based on AIC. The order of dropped variables was varied to ensure that final models were the most stable. Final models were validated by inspecting the Pearson residuals. McFadden’s pseudo-*R* ^2^ were calculated to assess the percentage of deviance explained by each model in relation to their respective null model^53^. Results were only presented for models with an *R* ^2^ > 0.1. All analyses were conducted in the software R^50^.

The final regression models were interpreted to identify the presence of additive and multiplicative effects as defined by Thrush *et al*.^43^, ignoring variables with p > 0.03 parameter estimates. Additive effects (i.e. when the combined effect of two stressors is equal to the sum of the individual effects) occurred when the effect of one explanatory variable was independent of the others in the model (e.g. expressed as + or − in the equation: y = a + b_1_x − b_2_x_2_). Multiplicative effects were identified by the presence in the final model of multiplicative terms (e.g. b_3_x_1_x_2_). Multiplicative effects were further interpreted as either synergistic (i.e. when the combined effect of two stressors is greater than the sum of the individual effects and both stressors operate in the same direction) or antagonistic (i.e. when the combined effect of two stressors is less than the sum of the individual effects and both stressors operate in the same direction) based on whether the sign of the parameter estimate for the multiplicative effects indicated that it worked to increase or decrease the effect of strongest variable identified by the model. A third type of multiplicative interaction, opposing synergistic, was defined when the combined effect of two stressors was greater than the sum of the individual effects but the stressors operated in opposite directions.

## Results

### Infauna and functional traits

One hundred and seventeen different taxa were found across the 75 sites sampled. Four of the modeled taxa dominated the samples (>1000 individuals in total); nut shells (*Linucula hartvigiana*), cockles (*Austrovenus stutchburyi*), brown anemones (*Anthopleura aureoradiata*) and wedge shells (*Macomona liliana*). Species diversity ranged from 9 to 39 taxa per site while mean abundance ranged from 8 to 223 individuals per core. None of the modelled taxa were found at the site with the highest sediment, nutrient and metal levels.

For the modelled traits, each trait was represented by between 7 and 97 taxa and present at more than 40 sites. Most of the traits were found across the entire stressor range for all three stressors, with the exception of sedentary and limited motility, simple hole or pit and tube habitat structures and attached living position traits, which had lower numbers of taxa (n = 7–21) representing the trait and were not found at the site that had the highest sediment, nutrient and metal levels.

### Physico-chemical variables

Sediments sampled within Tauranga Harbour encompassed a wide range of grain sizes (<0.1% to 76.4% silt and clay) but were predominantly sandy (51–100% sand) with relatively low organic content (most sites between 0.9–4.5% LOI; Table 2). Nutrient levels (140–1900 mg/kg TN; 51–580 mg/kg TP) were indicative of a slightly to moderately enriched estuary and metal concentrations were well below metal sediment quality guidelines used worldwide^54^. For example, the Australian and New Zealand Environment and Conservation Council^55^ Interim Sediment Quality Guidelines-low values, which provide thresholds for possible biological effects, are 65, 50 and 200 mg/kg for copper, lead and zinc, respectively. Chlorophyll-α generally ranged from 1100 to 16,000 µg kg^−1^. Sediment mud content, nutrients and metals were all higher in inner harbour areas compared with outer sites^23^.

**Table 2:** Key sediment and infauna variables for 75 sites in Tauranga Harbour (minimum, average and maximum values). Traits were modelled across all sites and maximum values for sites modelled for taxa are provided.

	Gravel	Sand	Silt/clay	LOI	TN	TP	Cu	Zn	Pb	Chl-*α*	*N*	*S*
	%	%	%	%	mg/kg	mg/kg	mg/kg	mg/kg	mg/kg	μg/kg		
Min	<0.1	23.7	<0.1	0.9	140	51	<1.0	<5.0	<1.0	210	29	10
Ave	1.7	84.3	13.2	2.9	481	169	1.0	17.4	2.6	6144	117	25
Max	14.6	100.0	76.4	10	1900	580	6.1	55.0	13.0	16000	333	39
Max modelled (taxa)	14.6	100.0	48.9	4.5	1000	340	3.0	55.0	5.6	16000	333	39

Notes: Gravel (>2 mm), sand (<2 mm and >63 μm), silt/clay (<63 μm), LOI = loss on ignition; TN = total nitrogen; TP = total phosphorous*;* Cu = copper; Zn = zinc; Pb = lead, Chl-*α* *= *chlorophyll-*a; N* = total abundance per core; *S* = total number of taxa per site.

### Taxon-specific responses to stressors

**Figure 2:**
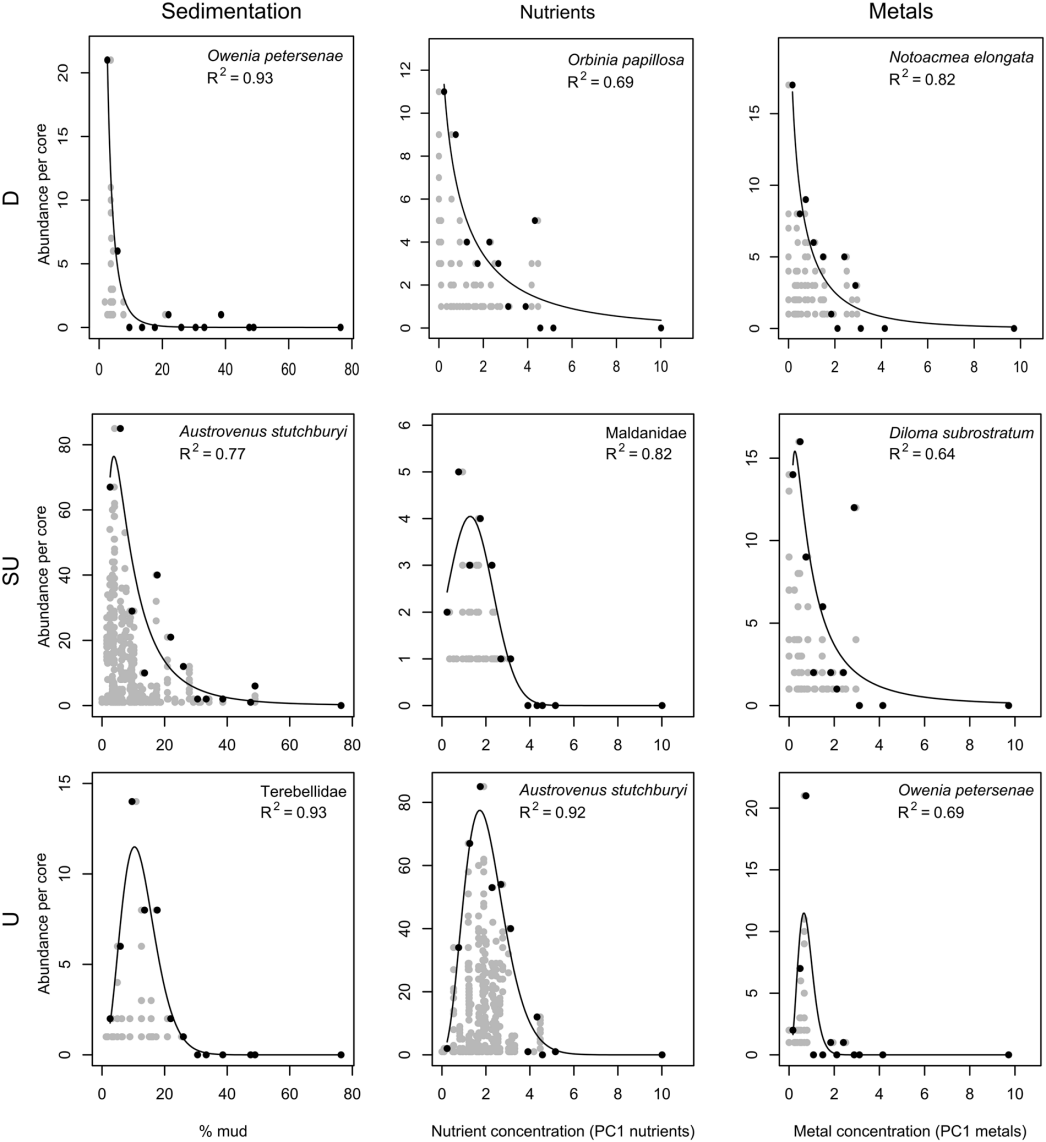
Representative plots of the relationship between individual taxa (as indicated) and sedimentation (left), nutrients (middle) and metal loading (right). Examples of decline responses (D; top row), skewed unimodal responses (SU; middle row) and unimodal responses (U; bottom row) are shown. Each grey point is a single core, with 10 cores taken from each of 75 sites. Black points show the binned data. The generalised linear model for maximum abundance of the binned data is shown.

All taxa displayed clear differences in abundance (*R* ^2^ ≥ 0.55) as a function of at least one of the pressure gradients: sedimentation, nutrient or metal loading (Table 3). The models revealed a variety of functional forms, indicating species-specific sensitivity to mud content, nutrients and/or metal loading (Fig. 2). We identified three types of responses; decline, unimodal and skewed unimodal. A decline response was defined as a decrease in abundance with increasing stressor values, a unimodal response was defined as a relatively large increase in abundance followed by an equivalent decrease and a skewed unimodal response was defined as a relatively small increase in abundance at the lower end of the stressor range followed by a decrease. In most cases, skewed unimodal responses were more similar to decline responses than unimodal responses, however, in all cases an initial increase in abundance was observed at the lower end of the stressor range.

**Table 3:** Summary of generalised linear models predicting maximum density for 20 macroinvertebrate taxa in response to sedimentation, nutrients and metal loading.

Taxa	Group	Sedimentation			Nutrients				Metal loading			
		*R* ^2^	Response	Optimum (%)	*R* ^2^	Response	Optimum) (mg/kg)		*R* ^2^	Response	Optimum (mg/kg)	
				Mud			TN	TP			Cu	Pb
*Anthopleura aureoradiata*	A	0.88	SU	2.6–7.3	0.56	U	300–505	110–175	0.37	D	0.3–0.5	1.3–1.6
*Arcuatula senhousia*	B	0.45	U	7.3–13.4	0.75	U	480–555	170–195	0.61	U	0.4–0.7	1.5–2.0
*Austrovenus stutchburyi*	B	0.77	SU	2.6–9.2	0.92	U	290–590	110–205	0.64	SU	0.4–0.8	1.3–2.2
*Linucula hartvigiana*	B	0.88	D	2.6–5.2	0.91	U	280–545	105–190	0.68	SU	0.4–0.9	1.3–2.5
*Macomona liliana*	B	0.45	SU	2.9–17.6	0.55	U	265–755	100–255	0.23	D	0.3–1.9	1.3–4.5
*Paphies australis*	B	0.90	D	2.6–3.2	0.95	U	370–470	135–165	0.97	D	0.3–0.4	1.3–1.4
*Zemysia zelandica*	B	0.63	U	4.8–11.5	0.67	U	420–540	150–190	0.54	U	0.7–1.2	2.0–3.1
*Halicarcinus cookii*	D	0.82	SU	2.6–12.3	0.43	U	265–590	105–204	0.63	SU	0.5–1.1	1.5–2.8
*Diloma subrostratum*	G	0.75	D	2.6–6.1	0.49	U	215–445	85–155	0.64	SU	0.4–0.8	1.3–2.1
*Micrelenchus huttonii*	G	0.49	SU	2.6–19.7	0.64	U	250–570	95–200	0.51	D	0.3–1.2	1.3–3.1
*Notoacmea elongata*	G	0.93	D	2.6–5.8	0.69	U	170–255	100–175	0.82	D	0.3–0.5	1.3–1.6
*Zeacumantus lutulentus*	G	0.87	D	2.6–3.5	0.18	U	210–625	85–215	0.55	U	0.4–0.9	1.3–2.4
*Zeacumantus subcarinatus*	G	0.98	D	2.6–3.7	0.63	U	280–490	105–170	0.87	U	0.4–0.7	1.3–2.0
*Hyboscolex longiseta*	P	0.74	U	8.6–24.2	0.54	U	325–685	120–235	0.41	U	0.4–1.1	1.4–2.9
*Magelona dakini*	P	0.98	D	2.6–3.2	0.99	U	305–440	110–155	Not significant			
*Maldanidae*	P	0.91	SU	2.7–8.9	0.82	SU	170–520	70–180	0.37	SU	0.4–0.8	1.3–2.3
*Orbinia papillosa*	P	0.77	D	2.6–5.8	0.69	D	170–255	70–100	0.61	D	0.3–0.5	1.3–1.6
*Owenia petersenae*	P	0.93	D	2.6–3.3	0.70	U	320–470	120–165	0.64	U	0.5–0.8	1.5–2.3
*Scoloplos cylindrifer*	P	0.79	D	2.6–9.3	0.39	U	185–470	75–165	0.54	D	0.4–0.5	1.3–1.6
Terebellidae	P	0.93	U	5.8–16.5	0.23	U	250–555	95–195	0.68	U	0.5–1.4	1.5–3.5

Only significant (p < 0.05) models are displayed. Taxonomic groups are: A = Anthozoa, B = Bivalva, D = Decapoda, G = Gastropoda, P = Polychaeta. Responses are: D = decline, SU = skewed unimodal, U = unimodal as represented in Fig. 2. Optimum range (upper 40% of the raw (non-zero) abundance data) indicates the stressor range over which taxa exhibit their highest abundances.

All species, except two, showed unimodal responses to nutrient loading, while all three types of responses were found for sedimentation and metal loading. No continuous increasing responses to the stressors were found for any of the taxa and only one model was non-significant. The bivalve *Macomona liliana* showed relatively high tolerance to all three stressors, with the greatest upper optimum value for nutrient and metal loading and the third highest for sedimentation, however, the variation explained by these models was relatively low (*R* ^2^ = 0.23–0.55). The polychaete *Magelona dakini*, the gastropod *Notoacmea elongata* and bivalve *Paphies australis* were often sensitive to stressors with low upper optimum values. The Supplementary Material details the models fitted to maximum abundance data for sedimentation, nutrients and metal loading.

### Sedimentation

For sedimentation, most models effectively explained variability in the distribution of maximum taxa abundance (*R* ^2^ > 0.60), except for three mollusc species (*Arcuatula senhousia*, *Macomona liliana* and *Micrelenchus huttonii, R* ^2^ < 0.50, Table 3). Most taxa (12) preferred sandier sediments and decreased in abundance in response to increasing mud. Responses of six taxa were classified as skewed unimodal (e.g., Fig. 2); with responses of the other four classified as unimodal.

Most taxa were distributed over the majority of the modelled sedimentation range (0–50% mud) with *Zemysia zelandica, Paphies australis, Halicarcinus cookii* and *Magelona dakini* showing more restrained distributions (0–15% mud; Table 3). Many of the modelled bivalves, gastropods and polychaetes showed a preference for sand (optima < 18% mud). The polychaete *Hyboscolex longiseta* showed the greatest tolerance for mud in terms of its maximum density (optima 9–25% mud) while the gastropod *Micrelenchus huttoni* had the widest optimum range (0–20% mud).

### Nutrients

For nutrients, most models effectively explained variability in the distribution of maximum abundance (*R* ^2^ > 0.50), except for the crab (*Halicarcinus cookii*), two gastropods (*Diloma subrostratum* and *Zeacumantus lutulentus*) and two polychaetes (*Scoloplos cylindrifer* and Terebellidae), for which only 18 to 49% of the variability could be explained (Table 3). All taxa, except two, exhibited unimodal responses (Fig. 2). The polychaete *Orbinia papillosa* exhibited a decline with increased nutrient loading while the Maldanidae family of polychaetes showed a skewed unimodal response.

Taxa distribution and optima in response to nutrient loading were species specific. For example, some bivalves (*Paphies australis*, *Arcuatula senhousia* and *Zemysia zelandica)* exhibited small distributional and optima ranges (optima TN 370–555 mg/kg; TP 135–195 mg/kg) while others (*Macomona liliana, Linucula hartvigiana* and *Austrovenus stutchburyi*) were less restricted (optima TN 265–760 mg/kg; TP 100–260 mg/kg; Table 3; Fig. 2). The optima for most taxa was at the lower end of the modelled nutrient range (TN 185–630 mg/kg; TP 75–215 mg/kg).

### Metal loading

For metal loading, most models effectively explained variability in the distribution of maximum abundance (*R* ^2^ > 0.50), except for *Anthopleura radiata*, *Macomona liliana*, *Hyboscolex longiseta* and Terebellidae, for which only 21 to 41% of the variability could be explained (Table 3). No relationship between metal concentrations and abundance was found for the polychaete *Magelona dakini*.

Many of the taxa were found across the majority of the modelled metal concentration range but, similar to sedimentation, *Zemysia zelandica, Paphies australis, Halicarcinus cookii and Magelona dakini* were restricted to less contaminated sites (Cu < 1.4, Pb < 3.6 mg/kg; Table 3). The optimum abundances for all taxa were at the less contaminated end of the range (Cu < 1.3, Pb < 3.2 mg/kg), with the exception of *Macomona liliana* whose optima encompassed the entire stressor range of the other taxa (Cu < 2.0, Pb < 4.6 mg/kg, *R* ^2^ = 0.23). Seven taxa declined in response to increasing sediment metal concentrations while another seven were classified as exhibiting a skewed unimodal response (Fig. 2). The remaining seven taxa displayed unimodal responses. Many of the responses to metal loading were similar to those found for sedimentation, and even where the response changed from decline to unimodal, as for *Owenia petersenae*, *Zeacumatus lutulentus* and *Zeacumantus subcarinatus*, the optimum abundance for both stressors was still at the lower end of the range.

### Functional responses to stressors

All functional traits displayed clear differences in abundance (*R* ^2^ > 0.50) as a function of at least one of the stressor gradients: sedimentation, nutrient or metal loading (Table 4). All traits showed a unimodal response to nutrient loading, while all three types of responses were found for sedimentation and metal loading. No continuous increasing responses to the stressors were found for any of the traits.

**Table 4:** Summary of generalised linear models predicting maximum density for 22 functional traits in response to sedimentation, nutrients and metal loading.

Trait	Group	Sedimentation			Nutrients				Metal loading			
		*R* ^*2*^	Response	Optimum (%)	*R* ^*2*^	Response	Optimum (mg/kg)		*R* ^*2*^	Response	Optimum (mg/kg)	
				Mud			TN	TP			Cu	Pb
Freely motile	M	0.67	D	2.6–13.8	0.46	U	230–850	90–285	0.49	D	0.3–1.2	1.3–3.1
Sedentary	M	0.74	SU	2.6–8.0	0.71	U	320–545	115–190	0.90	SU	0.4–0.6	1.3–1.8
Limited	M	0.88	D	2.6–5.3	0.89	U	270–575	100–200	0.70	SU	0.4–1.0	1.3–2.6
Deposit	F	0.70	D	2.6–7.8	0.71	U	255–750	100–255	0.41	SU	0.4–1.2	1.3–3.0
Suspension	F	0.84	D	2.6–7.5	0.86	U	290–540	110–190	0.83	D	0.3–0.5	1.3–1.6
Simple hole or pit	H	0.87	D	2.6–5.0	0.86	U	270–560	100–195	0.71	U	0.4–1.0	1.3–1.8
Permanent burrow	H	0.54	U	2.6–22.4	0.40	U	285–1065	105–355	0.41	D	0.3–6.0	1.3–13.3
Trough	H	0.93	D	2.6–4.5	0.58	U	250–575	95–200	0.77	U	0.4–0.8	1.3–2.2
Tube	H	0.53	U	6.3–12.3	0.61	U	440–575	155–200	0.89	U	0.4–0.6	1.4–1.9
Surface mixing	S	0.83	D	2.6–5.0	0.89	U	255–540	100–190	0.86	D	0.4–0.7	1.3–1.9
Deep-to-surface	S	0.54	D	2.6–14.8	0.26	U	245–965	95–325	0.54	D	0.4–0.7	1.3–2.1
Surface-to-deep	S	0.30	U	3.3–16.5	0.35	U	380–740	135–250	0.56	U	0.4–0.8	1.4–2.3
Small	BS	0.59	D	2.6–7.2	0.66	U	280–645	105–220	0.72	D	0.4–0.6	1.3–1.8
Medium	BS	0.75	SU	2.6–11.3	0.48	U	280–645	105–220	0.69	U	0.4–0.9	1.3–2.4
Large	BS	0.82	SU	2.6–11.7	0.86	U	240–645	90–220	0.68	D	0.4–1.0	1.3–2.6
Round/globulose	BF	0.88	D	2.6–7.5	0.93	U	265–525	100–180	0.84	D	0.4–0.7	1.3–1.9
Worm-shaped	BF	0.42	SU	2.6–15.4	0.35	U	320–790	115–790	0.53	U	0.4–0.9	1.3–2.5
Top 2 cm	L	0.94	D	2.6–4.8	0.51	U	250–625	95–215	0.67	U	0.4–0.9	1.3–2.5
Deeper than 2 cm	L	0.63	D	2.6–14.5	0.35	U	260–1065	100–355	0.52	D	0.3–0.9	1.3–2.4
Attached	L	0.94	D	2.6–5.7	0.83	U	290–470	110–165	0.81	D	0.3–0.4	1.3–1.5
Soft-bodied	BH	0.63	SU	2.6–13.9	0.59	U	320–705	115–240	0.74	SU	0.4–0.8	1.3–2.3
Calcified	BH	0.81	D	2.6–6.5	0.96	U	270–575	100–200	0.83	SU	0.4–0.7	1.3–2.1

**Figure 3:**
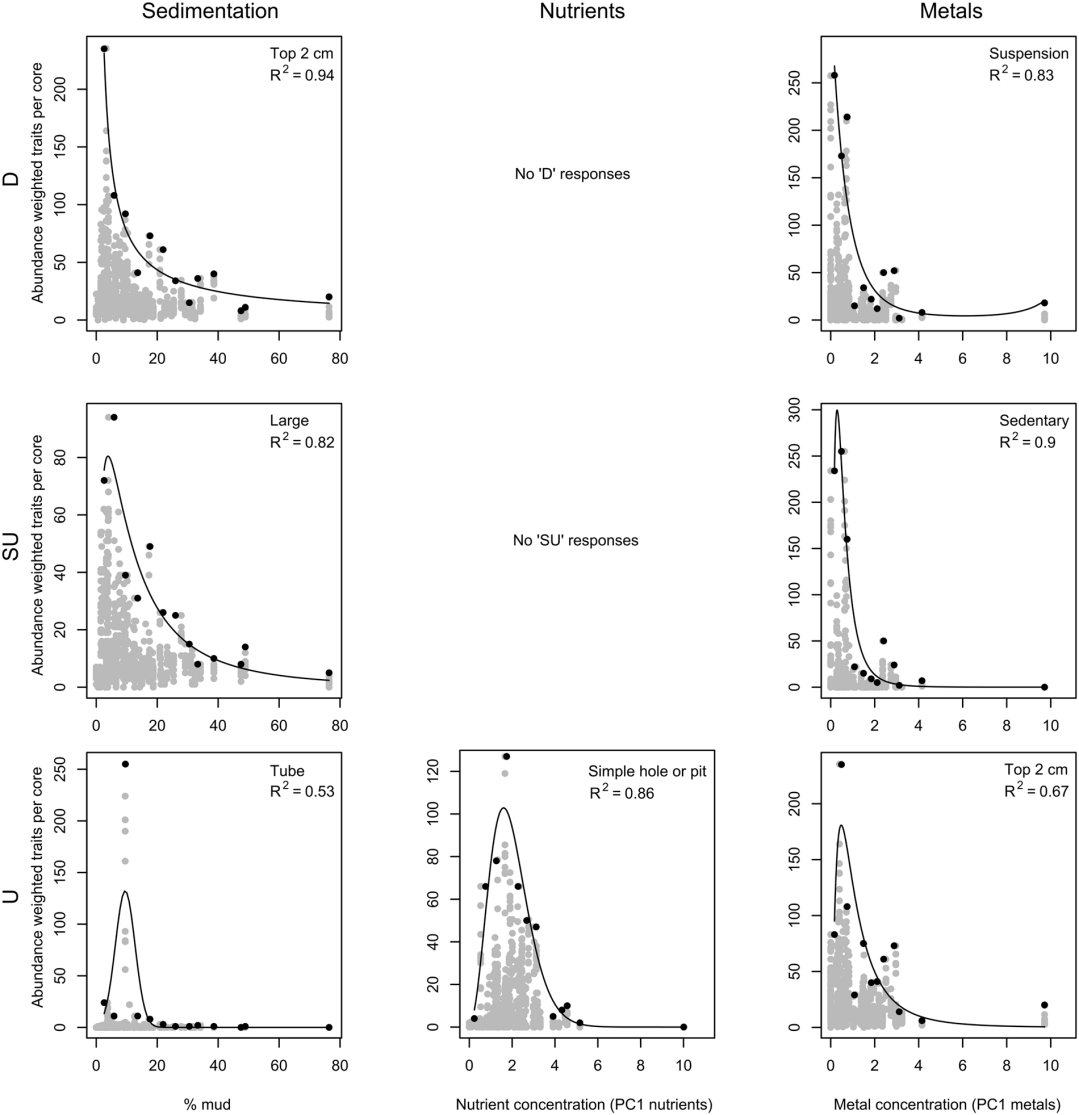
Representative plots of the relationship between individual traits (as indicated) and sedimentation (left), nutrients (middle) and metal loading (right). Examples of decline responses (D; top row), skewed unimodal responses (SU; middle row) and unimodal responses (U; bottom row) are shown. Each grey point is a single core, with 10 cores taken from each of 75 sites. Black points show the binned data. The generalised linear model for maximum abundance of the binned data is shown.

Only significant (p < 0.05) models are displayed. Functional trait categories are: M = motility, F = feeding, H = habitat structure, S = sediment particle movement, BS = body size, BF = body form, L = living position in the sediment, BH = body hardness. Responses are: D = decline, SU = skewed unimodal, U = unimodal as represented in Fig. 3. Optimum range (upper 40% of the raw (non-zero) abundance data) indicates the stressor range over which taxa exhibit their highest abundances.

Most traits were distributed across entire modelled stressor ranges, with the exception of the five traits that were not present at the most impacted site. On average, the optima for traits tended to be at higher stressor levels and across a broader range than for taxa. Permanent burrow habitat structure consistently showed the greatest tolerance to the three modelled stressors, however, the variation explained by these models was relatively low (*R* ^2^ = 0.40–0.54). Attached living position, round/globulose body form and suspension feeding traits were most sensitive to nutrients and metal loading while simple hole or pit habitat structure and surface mixing sediment particle movement traits were most sensitive to sedimentation. The Supplementary Material details the models fitted to maximum trait abundance data for sedimentation, nutrients and metal loading.

#### Sedimentation

For sedimentation, most models effectively explained variability in the distribution of maximum trait abundance (*R* ^2^ > 0.50), except for surface-to-deep sediment movement and worm-shaped body form traits, for which only 30% and 42% of the variability in trait abundance could be explained, respectively (Table 4). Most of the traits (14) declined in response to increasing sedimentation (Fig. 3). Responses of five traits were classified as skewed unimodal; with responses of three traits (surface-to-deep sediment movement, permanent burrows and tube structures) classified as unimodal. The optima for most traits was less than 16% mud content with permanent burrow habitat structure showing the greatest tolerance for mud in terms of maximum density (optima < 23% mud; (Table 4).

#### Nutrients

For nutrients, most models effectively explained variability in the distribution of maximum trait abundance (*R* ^2^ > 0.50), except for freely motile, deep-to-surface and surface-to-deep sediment movement, medium body size, worm-shaped body form and deeper than 2 cm living position traits, for which only 26 to 48% of the variability in trait abundance could be explained. All traits exhibited unimodal responses (Fig. 3). The optima for most traits was less than 955 mg/kg TN and 320 mg/kg TP, with permanent burrow habitat structure, living positions deeper than 2 cm and deep-to-surface sediment movement showing the greatest tolerance for nutrients in terms of maximum density (optima TN 245–1065 mg/kg, TP 95–355 mg/kg; Table 4).

#### Metal loading

For metal loading, most models effectively explained variability in the distribution of maximum trait abundance (*R* ^2^ > 0.50), except for freely motile, deposit feeder, and permanent burrow habitat structure traits, for which only 41 to 49% of the variability in trait abundance could be explained. As for sedimentation, most of the traits (10) declined in response to increasing metal loading (Fig. 3). Responses of five traits were classified as skewed unimodal with responses of seven classified as unimodal responses. Many of the responses to metal loading were similar to those found for sedimentation, however, for some traits, such as permanent burrow habitat structure and top 2 cm living position, the response was different (Fig. 3). The optima for most traits was less than 1.3 mg/kg Cu and 3.1 mg/kg Pb, with permanent burrow habitat structure, deposit feeders and freely motile traits showing the greatest tolerance for metal loading in terms of maximum density (optima Cu < 6.1 mg/kg, Pb < 13.4 mg/kg; Table 4).

### Multiple stressors

**Figure 4:**
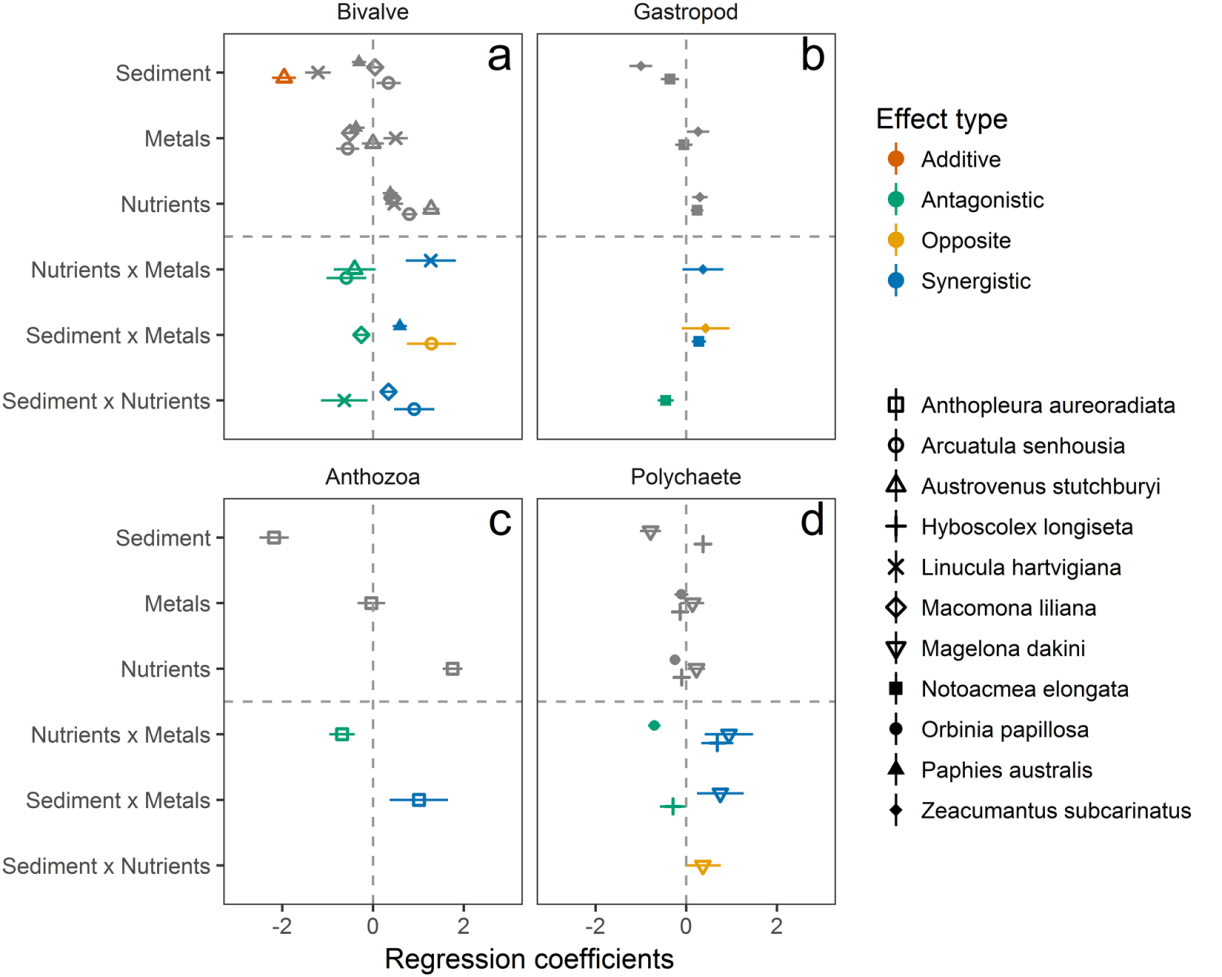
Estimated regression coefficients (±CI) for each taxa group: (**a**) bivalves, (**b**) gastropods, (**c**) anthozoans and (**d**) polychaetes in response to stressors: sedimentation (% mud), nutrients (PC1 nutrients) and metal loading (PC1 metals). Additive effects (red) occur when the effect of one stressor is independent of others in the model and are either negative (left) or positive (right). Multiplicative effects occur when there is an interaction effect and these are antagonistic (green) when individual stressors act in the same direction and there is a negative interaction effect, synergistic (blue) when individual stressors act in the same direction and there is a positive interaction effect, and opposing synergistic (yellow) when individual stressors act in opposing directions and there is a positive interaction effect. Grey symbols indicate single stressor effects.

**Figure 5:**
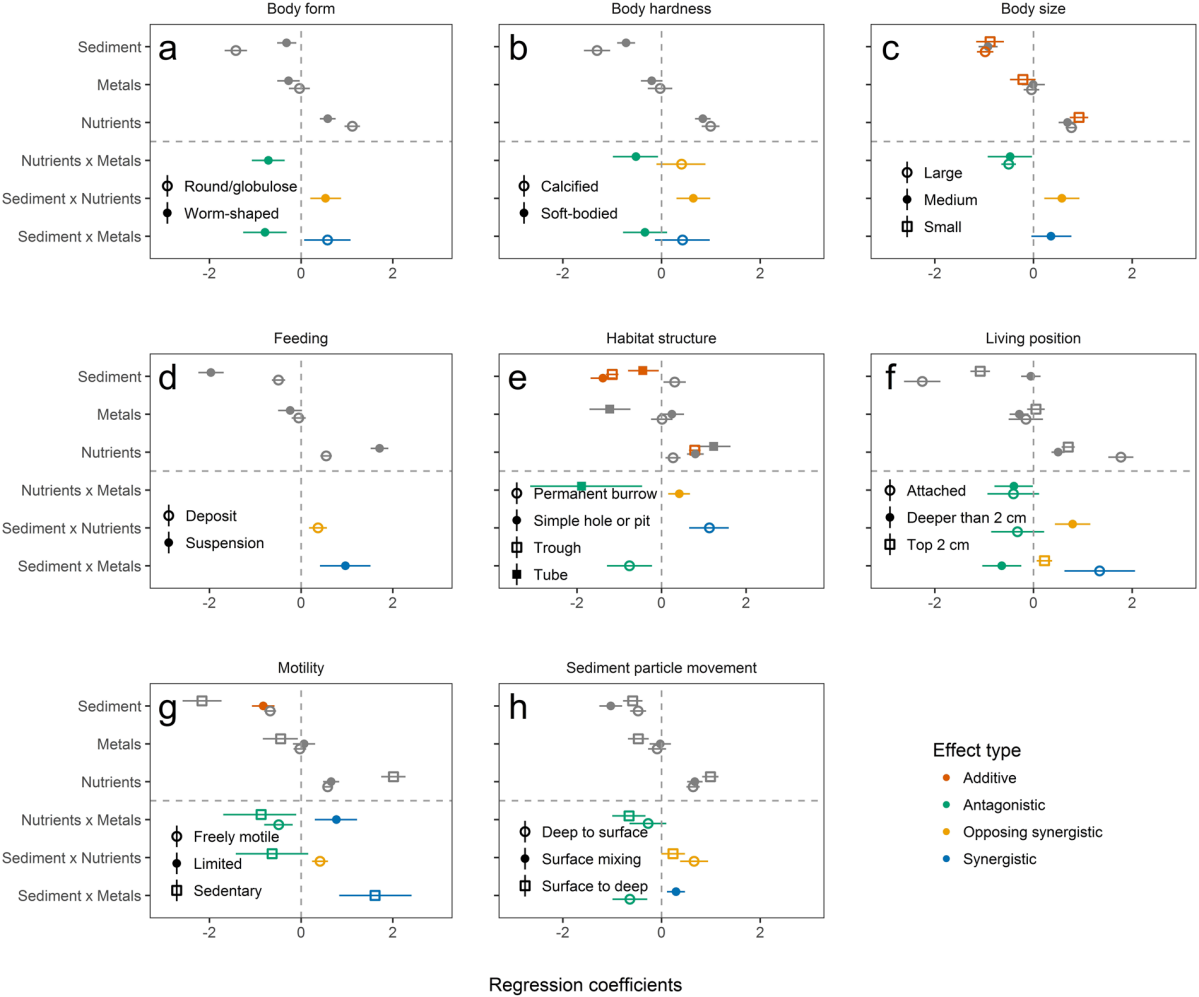
Estimated regression coefficients (±CI) for each functional trait: (**a**) body form, (**b**) body hardness, (**c**) body size, (**d**) feeding, (**e**) habitat structure, (**f**) living position, (**g**) motility and (**h**) sediment particle movement in response to stressors: sedimentation (% mud), nutrients (PC1 nutrients) and metal loading (PC1 metals). Additive effects (red) occur when the effect of one stressor is independent of others in the model and are either negative (left) or positive (right). Multiplicative effects occur when there is an interaction effect and these are antagonistic (green) when individual stressors act in the same direction and there is a negative interaction effect, synergistic (blue) when individual stressors act in the same direction and there is a positive interaction effect, and opposing synergistic (yellow) when individual stressors act in opposing directions and there is a positive interaction effect. Grey symbols indicate single stressor effects.

Final models relating taxa and trait abundances to variations in sedimentation, nutrients and metal loading can be found in the Supplementary Material and summary plots are displayed in Fig. 4 and Fig. 5. Over all significant models (*R* ^2^ > 0.1), we identified 10 additive responses (eight negative, two positive), 26 antagonistic interactions, 19 synergistic interactions and 14 opposing synergistic interactions. The total deviance explained by the models was generally low (*R* ^2^ range = 0.14 to 0.49).

For both taxa and functional traits, single stressor effects were usually negative for sedimentation, positive for nutrients and negative for metal loading. Multiplicative effects were identified for all three pairs of stressor combinations. The interaction between metal loading and sedimentation was most often synergistic (52% of multiplicative interactions) but antagonistic (33%) and opposing synergistic (14%) interactions were also observed. Where nutrients and sedimentation interacted, sedimentation often had less of a negative effect when nutrients were present than predicted by an additive model (i.e. opposing synergistic, 56% of multiplicative interactions) but antagonistic (25%) and synergistic (19%) interactions were also observed. For functional traits, interactions between nutrients and metal loading were usually antagonistic (79%) but equal numbers of synergistic and antagonistic interactions were found for this stressor combination for taxa. All taxa and traits were involved in multiplicative interactions, with the exception of trough habitat structure and small body size.

## Discussion

### Response to stressors

Maximum abundance models of the response of estuarine organisms and functional traits to key anthropogenic stressors were developed. Taxa and traits were either sensitive to elevated sedimentation, nutrients or metal loading at all levels, or sensitive to these stressors beyond a critical point. For both taxa and traits, unimodal responses were almost always observed in response to nutrients, while declines or skewed unimodal responses were most often observed in response to sedimentation and metal loading. In most cases, the responses to metal loading were similar to those found for sedimentation, and even where the response changed from decline to unimodal the optimum abundance for both stressors was still at the lower end of the range. The occurrence of unimodal responses to sedimentation or metal loading may arise from indirect effects that occur when those taxa are released from competition or other interspecific interactions due to the decline of their more sensitive counterparts^18^. For example, taxa living attached to other organisms or deeper than 2 cm declined in response to increasing metal loading, which may have allowed taxa that live in the top 2 cm to show an initial increase in abundance. Beyond a certain point, however, these taxa may also become intolerant of the stressor resulting in a unimodal response.

The prevalence of declining and skewed unimodal responses (78% of responses to sedimentation and metal loading) is concerning given the relatively low stressor levels in this study. Declines in abundance were observed for a range of species including those with traits that have important implications for ecosystem functioning, such as large body size, suspension feeding and freely motile taxa. Large taxa living within the sediments provide a disproportionate contribution to ecosystem functioning through bioturbation, modification of boundary layers and the provision of food for other consumers^56,57^. Freely motile taxa also contribute to bioturbation, which in turn affects nutrients and oxygen fluxes^58^, while the loss of suspension feeders could have implications for benthic-pelagic coupling and water clarity^59,60^.

Many of the taxa were distributed over the majority of the modeled stressor ranges but in general optimum abundances were at the lower end of the ranges for all three stressors, highlighting the vulnerability of estuaries to increasing stressor loads. The bivalve *Macomona liliana* and the permanent burrow habitat structure trait were consistently tolerant of all three stressors. Although the variation explained by these models was relatively low, thirty percent of the taxa (or closely related taxa) contributing to the permanent burrow trait group were assigned to the two most tolerant eco-groups (IV & V) by AMBI^61^ and Robertson *et al*.^30^ compared with less than eight percent of taxa contributing to more sensitive traits suggesting permanent burrow may be a trait that confers tolerance to stressors. However, the relatively sensitive eco-group (II) assigned to *Macomona liliana* ^30^ implies further research may be required to ascertain the stress response for this species. Sensitive taxa included the polychaete *Magelona dakini*, the gastropod *Notoacmea elongata* and the bivalve *Paphies australis*, which is consistent with the relatively sensitive eco-groups assigned to these taxa by the AMBI^61^ and Robertson^30^. Attached living position, round/globulose body form and suspension feeding traits were most sensitive to nutrients and metal loading and simple hole or pit habitat structure and surface mixing sediment particle movement traits were most sensitive to sedimentation. In agreement with this, seventy to eighty-five percent of the taxa that exhibited these traits were in the two most sensitive eco-groups I & II^30,61^. The finding that suspension feeding traits were most sensitive to nutrients may seem surprising because suspension feeders utilize pelagic microorganisms and therefore might be expected to be uncoupled from sediment nutrients. However in a New Zealand context, it has been found that suspension feeders frequently feed on resuspended benthic algae^62^ and this is thought to be particularly important for intertidal species.

At the 40% cut off, the optima for traits tended to be at higher stressor levels and across a broader range than for taxa, reflecting the redundancy inherent within functional groups and suggesting taxa may be more sensitive for detecting changes in ecosystem health. Declines in the abundances of a few taxa may not be of concern if the functional traits are still present and performing well, however, the loss of taxa is likely to lead to a reduction in ecosystem resilience even if functionality is maintained. As the sensitivity of traits is highly dependent on how traits are defined, different traits would need to be tested at different study sites in order to reach more generic conclusions about the relative sensitivity of taxa and traits.

Using a more stringent cut off of 20%, the optimum ranges and upper optimum values were not always broader and wider for traits with respect to sedimentation and metal loading, emphasizing that traits showed more declining responses to these stressors than taxa. For both taxa and traits, declining responses were most common for sedimentation and metal loading, therefore, any unimodal response exhibited by an individual taxa would likely be subsumed when it was included in a trait response resulting in more declining responses for traits.

In general, model comparisons of the relative sensitivity of different taxa revealed wide variations in response to changes in environmental conditions. For example, *Hyboscolex longiseta* and Terebellidae showed a unimodal response to sedimentation whereas other polychaetes simply declined in abundance with increasing mud. Similarly, some bivalves had narrow distributions and optima in response to nutrients (e.g. *Paphies australis, Zemysia zelandica*) while others were found across wider ranges of concentrations (e.g. *Austrovenus stutchburyi* and *Macomona liliana*). Other studies have also found species-specific differences in habitat preference^30,63,64^ and these differences have important implications for conclusions drawn from studies of low taxonomic resolutions, or aggregative indices of ecological change of ecosystem ‘health’.

This study supports the general findings from previous research^30,63–66^ of strong changes in benthic macrofauna distribution in relation to percentage mud, with important implications for assessing long term responses of communities to habitat change. Increased sediment, either deposited on the seafloor via catastrophic events^12^ or chronic sediment suspended in the water column, can negatively impact organisms (i.e. via burial, scour, inhibiting settlement, decreasing filter feeding efficiency, decreasing light penetration) and lead to reductions in diversity, abundance and the loss of functionally important species^67^. Optimum abundance of most taxa and traits documented in this study were less than 18% mud content. For seventy percent of our modelled taxa, the estimated optimum mud content was comparable to at least one other study^30,63,64,68^, where corresponding estimates were available. Taxon-specific responses to mud were often variable across studies, however, in general, Robertson *et al*.’s^30^ optimum mud ranges were higher than others, possibly reflecting the wider geographical area sampled, or potential differences in geo-chemical structure of the muds. For example, the optimum upper limit of 9.2% mud for *Austrovenus stutchburyi* recorded in this study was comparable to 10.0–11.3% estimated by Norkko *et al*.^68^, Thrush *et al*.^64^ and Anderson *et al*.^63^ but lower than the 44.9% estimated by Robertson *et al*.^30^.

The present study extends current knowledge by examining the response of key macrofauna to two other important coastal stressors; nutrient and metal loading. Taxa responses to nutrients were almost always unimodal responses. Low levels of nutrient enrichment in estuarine and coastal environments can have a positive effect on the benthos due to improved primary productivity, and therefore food availability^69^. Beyond a critical point, however, many studies have documented that excessive nutrient discharges can lead to accelerated eutrophication of coastal environments and over enrichment impacts^15,69^. Optimum abundances of most taxa and traits documented in this study were at relatively low nutrient concentrations (taxa: TN 185–630 mg/kg, TP 75–215 mg/kg; traits: TN 245–955 mg/kg, TP 95–320 mg/kg), suggesting communities will be sensitive to increasing nutrient loads.

As for metals, the optimum abundances of most taxa and traits were at metal concentrations (Cu < 1.3, Pb  < 3.2 mg/kg) that are well below current guidelines used to manage metal contamination worldwide^54^. Multivariate analysis of the entire community showed that communities at the lower end of the metal loading gradient were, on average 71% dissimilar to those at the most contaminated sites providing evidence of benthic community change across this low-level metal gradient^70^. Hewitt *et al*.^71^ also observed declines in estuarine infauna at relatively low metal concentrations (6.5–9.3 mg/kg Cu,18.8–19.4 mg/kg Pb) and a strong gradient of community change across a low metal concentration contaminant gradient. Similarly, along the Norwegian coast, concentrations as low as 2.1 mg/kg for copper and 8.6 mg/kg for lead caused changes in faunal communities^72^. These studies suggest that current sediment quality guidelines, which have higher thresholds, may not adequately protect coastal ecosystems from the adverse effects of contaminants.

There is increasing evidence that sediment quality guidelines developed from reviews of laboratory dose-response experiments are higher than those derived from field surveys^72,73^. The present study used declines in the abundance of taxa or traits as an endpoint whereas less conservative mortality-based endpoints are more common in laboratory studies^74^. Additionally, field surveys incorporate the simultaneous effects of multiple stressors^73^ (other metals as well as non-metal stressors), which can result in some organisms showing increased responses to metal contamination^43,75^. A review by Norwood *et al*.^76^ found that additive or more than additive responses (synergistic, potentiation, coalitive) were documented in 56% of studies addressing impacts from metal mixtures, suggesting that field studies such as ours, which assess species responses to more than one metal (e.g. copper and lead) may observe responses at lower metal concentrations than single-contaminant studies. Field surveys also allow for regional differences and variability^57,77,78^ and the presence of different species, indirect effects arising from biological interactions or sub-lethal effects^18,79–81^, and the differential susceptibility of life stages.

However, determining the relationship between species abundances and metal loading using field surveys results in high unexplained variability and predictions are limited to the effects that occur within the gradient of already existing contamination^71^. The particularly low optimum abundance values derived in this study for metal loading may be partially attributable to low levels of metal contamination in the study area and further research exploring taxa and trait responses over a greater gradient of metal loading would be required to more accurately predict optimum ranges for these taxa and traits.

### Multiple stressors

In agreement with other multiple stressor studies^1,2,43^, our results showed that multiplicative effects were more common than additive ones, with multiplicative effects identified for most taxa and traits modeled. The multiplicative effect of sedimentation with metal loading was most often synergistic suggesting that the negative effects of metal loading may exacerbate the impact of fine sediments. Thrush *et al*.^43^ also found synergistic interactions to be common between sedimentation (percentage mud) and individual metals. The negative effects of sedimentation and metal loading often became progressively less with increasing nutrients suggesting that the positive effects of low levels of nutrient enrichment may help to offset the negative effects of increased mud and contaminants. The prevalence of opposing synergistic interactions highlights the importance of considering stressor direction when interpreting interactions between multiple stressors^82^.

A review of multiple stressor effects based on 171 studies found the overall interaction effect across all of the studies was synergistic, but interaction type varied in relation to response level, trophic level and specific stressor pair^1^. Crain further noted that the addition of a third stressor changed the interaction effects significantly where it doubled the number of synergistic interactions. As most of Crain’s review studies were conducted in laboratories, where stressor effects can be carefully isolated, this suggests that synergies may be common in nature where more than two stressors generally coexist. Laboratory studies are important as they provide the mechanistic understanding of how and why interactions occur. However, direct transfer to the field can be confounded by the variety of communities (and their sensitivities) that exist in the field, differing temporal and spatial scales of studies^83^, and the presence of other stressors which could alter the direction and intensity of laboratory observed interactions (e.g. hydrodynamics, temperature variations). Crain concluded that although pollutants can affect communities and ecosystems, their effects can be reinforced when other pressures or stressors (i.e. nutrient inputs, habitat loss, hypoxia, etc.) are present. We would concur with these conclusions where we also found that, with multiple stressors, synergistic interactions were high, although interaction type varied by stressor pair and response level (i.e. species or functional trait).

It is important to recognise that the patterns described in this study are correlative and there may be other factors that may also be influencing macrofaunal assemblages. In general, collinearity between stressors can limit the ability to interpret multiplicative effects. Although sediments and metals often covary, previous studies have observed that collinearity between fine sediments (<63um) and metal concentrations were not sufficient to prevent partialling out of effects on macrofaunal communities^23,43,84^. Further manipulative experiments, however, would be required to prove causality. The predominance of multiplicative interaction effects on these important estuarine habitats highlights the complex nature of multiple stressor responses and emphasizes the importance of considering the range of stressors acting on the marine environment.

### Conclusions and further recommendations

Sedimentation, nutrients and metal loading are recognised as major stressors to coastal and estuarine systems, yet the lack of statistical models for marine soft-sediment ecosystems limits our predictive ability and hence management^26^. This study documents levels of sedimentation, nutrients and metal loading beyond which losses of species abundance and key ecosystem functions occur and provides insights into the nature and magnitude of multiple stressor effects in estuaries. The prevalence of multiplicative interactions, particularly synergistic effects between sedimentation and metal loading, observed in this study suggests that single stressor studies and policies that overlook the influence of concurrent stressors reduce the likely success of management outcomes and should be replaced by more strategic thinking, focusing on combinations of stressors shown to interact synergistically. However, our results suggest that key species and biodiversity per se may be more at risk than ecosystem services, with the abundances of traits less affected than taxa by changes in stressor levels. The context dependent and stressor specific nature of interactions also highlights the importance of experimental ecology to elucidate the mechanisms behind these interactions and inform strategies for management and conservation of our coastal and marine areas.

## Electronic supplementary material

Dataset 1
