# Supplementary material for: Multiple stressor effects on marine infauna: responses of estuarine taxa and functional traits to sedimentation, nutrient and metal loading
Source: Sci Rep. 2017 Sep 20;7:12013. doi: 10.1038/s41598-017-12323-5 (PMC5607226; doi:10.1038/s41598-017-12323-5)
Supplement: Supplementary file 1 — Dataset 1 [file 41598_2017_12323_MOESM1_ESM.doc]

**Supplementary Material for:**

Multiple stressor effects on marine infauna: responses of estuarine taxa and functional traits to sedimentation, nutrient and metal loading.

Ellis J.I. ^a,b*^, Clark D.^a^, Atalah J.^a^, Jiang W. ^a^, Taiapa C.^c^, Patterson M. ^d^, Sinner J.^a^, Hewitt J.^e^,

^a^ Cawthron Institute, Private Bag 2, Nelson 7042, New Zealand

^b^ King Abdullah University of Science and Technology (KAUST), Red Sea Research Center, Division of Biological and Environmental Science and Engineering, Thuwal 23955-6900, Saudi Arabia

^c^ Manaaki Te Awanui, 234a Waihi Rd, Tauranga 3110, New Zealand

^d^ Massey University, School of People Environment and Planning, Private Bag 11222, Palmerston North 4442, New Zealand

^e^ National Institute of Water and Atmospheric Research, PO Box 11115, Hillcrest, Hamilton 3216, New Zealand

Supplementary material

- Increase responses (continuous increase in abundance with increasing stressor values) were not observed for any taxa or functional traits.
- Decline responses were defined as a decrease in abundance with increasing stressor values.
- Skewed unimodal responses were defined as a relatively small increase in abundance at the lower end of the stressor range followed by a decrease.
- Unimodal responses were defined as a relatively large increase in abundance followed by an equivalent decrease.

SM-1. Summary of generalised linear models predicting maximum density for 20 macroinvertebrate taxa in response to sedimentation. Taxonomic groups are: A = Anthozoa, B = Bivalva, D = Decapoda, G = Gastropoda, P = Polychaeta. Responses are: D = decline, SU = skewed unimodal, U = unimodal as represented in Figure 2. Optimum range indicates the stressor (% mud) range over which taxa exhibit their highest abundances. Distribution range indicates the stressor range where at least one individual occurs.

| *Taxa* | *Group* | *Predictor*  *transformation* | *Polynomial*  *order* | *Null*  *deviance* | *R^2^* | *p value* | *Response* | *Optimum* | *Distribution* |
| --- | --- | --- | --- | --- | --- | --- | --- | --- | --- |
| *Anthopleura aureoradiata* | A | Log | 2 | 51792.75 | 0.88 | <0.001 | SU | 2.6-7.3 | <0.1-48.9 |
| *Arcuatula senhousia* | B | Log | 2 | 59362.92 | 0.45 | <0.001 | U | 7.3-13.4 | 2.6-25.4 |
| *Austrovenus stutchburyi* | B | Log | 2 | 19035.70 | 0.77 | <0.001 | SU | 2.6-9.2 | <0.1-48.9 |
| *Linucula hartvigiana* | B | Log | 1 | 17984.46 | 0.88 | <0.001 | D | 2.6-5.2 | <0.1-48.9 |
| *Macomona liliana* | B | Log | 2 | 2339.94 | 0.45 | <0.001 | SU | 2.9-17.6 | <0.1-48.9 |
| *Paphies australis* | B | Log | 1 | 10982.79 | 0.90 | <0.001 | D | 2.6-3.2 | 1.5-12.6 |
| *Zemysia zelandica* | B | None | 2 | 1886.33 | 0.63 | <0.001 | U | 4.8-11.5 | 1.5-15.1 |
| *Halicarcinus cookii* | D | None | 2 | 1514.44 | 0.82 | <0.001 | SU | 2.6-12.3 | 1.5-15.7 |
| *Diloma subrostratum* | G | Square root | 1 | 3582.62 | 0.75 | <0.001 | D | 2.6-6.1 | 1.5-48.9 |
| *Micrelenchus huttonii* | G | None | 2 | 1621.65 | 0.49 | <0.001 | SU | 2.6-19.7 | 1.5-27.9 |
| *Notoacmea elongata* | G | Square root | 1 | 3364.18 | 0.93 | <0.001 | D | 2.6-5.8 | <0.1-38.6 |
| *Zeacumantus lutulentus* | G | Log | 2 | 8896.73 | 0.87 | <0.001 | D | 2.6-3.5 | <0.1-48.9 |
| *Zeacumantus subcarinatus* | G | Log | 1 | 15535.34 | 0.98 | <0.001 | D | 2.6-3.7 | 1.3-48.9 |
| *Hyboscolex longiseta* | P | None | 2 | 1497.78 | 0.74 | <0.001 | U | 8.6-24.2 | 1.4-34.2 |
| *Magelona dakini* | P | Log | 1 | 17757.26 | 0.98 | <0.001 | D | 2.6-3.2 | 0.6-12.6 |
| Maldanidae | P | Log | 2 | 1218.86 | 0.91 | <0.001 | SU | 2.7-8.9 | 0.6-38.6 |
| *Orbinia papillosa* | P | Square root | 1 | 2697.50 | 0.77 | <0.001 | D | 2.6-5.8 | 0.6-47.5 |
| *Owenia petersenae* | P | Square root | 1 | 9062.67 | 0.93 | <0.001 | D | 2.6-3.3 | 1.8-38.6 |
| *Scoloplos cylindrifer* | P | None | 1 | 3635.93 | 0.79 | <0.001 | D | 2.6-9.3 | <0.1-48.9 |
| Terebellidae | P | Square root | 2 | 2831.26 | 0.93 | <0.001 | U | 5.8-16.5 | 1.3-25.4 |

SM-2. Summary of generalised linear models predicting maximum density for 20 macroinvertebrate taxa in response to nutrients. Taxonomic groups are: A = Anthozoa, B = Bivalva, D = Decapoda, G = Gastropoda, P = Polychaeta. Responses are: D = decline, SU = skewed unimodal, U = unimodal as represented in Figure 2. Optimum range indicates the stressor (PC1 nutrients) range over which taxa exhibit their highest abundances. Distribution range indicates the stressor range where at least one individual occurs.

| *Taxa* | *Group* | *Predictor*  *transformation* | *Polynomial*  *order* | *Null*  *deviance* | *R^2^* | *p value* | *Response* | *Optimum* | *Distribution* |
| --- | --- | --- | --- | --- | --- | --- | --- | --- | --- |
| *Anthopleura aureoradiata* | A | Square root | 2 | 40732.89 | 0.56 | <0.001 | U | 1.0-2.2 | 0-5.2 |
| *Arcuatula senhousia* | B | Log | 2 | 75673.71 | 0.75 | <0.001 | U | 2.1-2.5 | 1.1-3.9 |
| *Austrovenus stutchburyi* | B | Square root | 2 | 12221.16 | 0.92 | <0.001 | U | 0.9-2.7 | 0-5.2 |
| *Linucula hartvigiana* | B | Square root | 2 | 16592.39 | 0.91 | <0.001 | U | 0.9-2.5 | 0-4.6 |
| *Macomona liliana* | B | Square root | 2 | 1910.88 | 0.55 | <0.001 | U | 0.8-3.7 | 0-5.2 |
| *Paphies australis* | B | None | 2 | 7680.36 | 0.95 | <0.001 | U | 1.4-2.0 | 0.9-2.8 |
| *Zemysia zelandica* | B | None | 2 | 2662.60 | 0.67 | <0.001 | U | 1.7-2.4 | 1.1-2.4 |
| *Halicarcinus cookii* | D | None | 2 | 1124.97 | 0.43 | <0.001 | U | 0.9-2.7 | 0.5-2.6 |
| *Diloma subrostratum* | G | Log | 2 | 4236.58 | 0.49 | <0.001 | U | 0.5-1.9 | 0.5-3.9 |
| *Micrelenchus huttonii* | G | Log | 2 | 1372.45 | 0.64 | <0.001 | U | 0.7-2.6 | 0.5-4.5 |
| *Notoacmea elongata* | G | Log | 2 | 3036.31 | 0.69 | <0.001 | U | 0.8-2.2 | 0-4.5 |
| *Zeacumantus lutulentus* | G | Square root | 2 | 6904.23 | 0.18 | <0.001 | U | 0.5-2.9 | 0-5.2 |
| *Zeacumantus subcarinatus* | G | Square root | 2 | 12362.48 | 0.63 | <0.001 | U | 0.9-2.1 | 0-4.5 |
| *Hyboscolex longiseta* | P | Square root | 2 | 1558.88 | 0.54 | <0.001 | U | 1.2-3.3 | 0.1-3.9 |
| *Magelona dakini* | P | None | 2 | 15754.49 | 0.99 | <0.001 | U | 1.0-1.8 | 0.5-2.8 |
| Maldanidae | P | None | 2 | 715.24 | 0.82 | <0.001 | SU | 0.2-2.3 | 0.4-3.1 |
| *Orbinia papillosa* | P | Square root | 1 | 1179.14 | 0.69 | <0.001 | D | 0.2-0.7 | 0-4.5 |
| *Owenia petersenae* | P | None | 2 | 5955.37 | 0.70 | <0.001 | U | 1.1-2.0 | 0.4-3.1 |
| *Scoloplos cylindrifer* | P | Log | 2 | 3188.02 | 0.39 | <0.001 | U | 0.3-2.0 | 0.5-3.3 |
| Terebellidae | P | Log | 2 | 4027.25 | 0.23 | <0.001 | U | 0.7-2.5 | 0-3.9 |

SM-3. Summary of generalised linear models predicting maximum density for 20 macroinvertebrate taxa in response to metals. Taxonomic groups are: A = Anthozoa, B = Bivalva, D = Decapoda, G = Gastropoda, P = Polychaeta. Responses are: D = decline, SU = skewed unimodal, U = unimodal as represented in Figure 2. Optimum range indicates the stressor (PC1 metals) range over which taxa exhibit their highest abundances. Distribution range indicates the stressor range where at least one individual occurs.

| *Taxa* | *Group* | *Predictor*  *transformation* | *Polynomial*  *order* | *Null*  *deviance* | *R^2^* | *p value* | *Response* | *Optimum* | *Distribution* |
| --- | --- | --- | --- | --- | --- | --- | --- | --- | --- |
| *Anthopleura aureoradiata* | A | Square root | 1 | 42130.22 | 0.37 | <0.001 | D | 0.2-0.5 | 0-4.2 |
| *Arcuatula senhousia* | B | Log | 2 | 76466.67 | 0.61 | <0.001 | U | 0.3-0.8 | 0.3-2.5 |
| *Austrovenus stutchburyi* | B | Log | 2 | 15344.21 | 0.64 | <0.001 | SU | 0.2-0.9 | 0-4.2 |
| *Linucula hartvigiana* | B | Log | 2 | 13934.47 | 0.68 | <0.001 | SU | 0.2-1.2 | 0-3.2 |
| *Macomona liliana* | B | None | 2 | 1721.62 | 0.23 | <0.001 | D | 0.2-2.8 | 0-4.2 |
| *Paphies australis* | B | Square root | 1 | 10523.95 | 0.97 | <0.001 | D | 0.2-0.2 | 0-2.0 |
| *Zemysia zelandica* | B | None | 2 | 1880.24 | 0.54 | <0.001 | U | 0.7-1.6 | 0-1.5 |
| *Halicarcinus cookii* | D | None | 2 | 1493.01 | 0.63 | <0.001 | SU | 0.4-1.4 | 0-1.5 |
| *Diloma subrostratum* | G | Log | 2 | 3579.14 | 0.64 | <0.001 | SU | 0.2-0.9 | 0-3.0 |
| *Micrelenchus huttonii* | G | None | 2 | 1323.47 | 0.51 | <0.001 | D | 0.2-1.6 | 0-3.0 |
| *Notoacmea elongata* | G | Square root | 1 | 3702.77 | 0.82 | <0.001 | D | 0.2-0.4 | 0-3.0 |
| *Zeacumantus lutulentus* | G | Log | 2 | 7636.53 | 0.55 | <0.001 | U | 0.2-1.1 | 0-4.2 |
| *Zeacumantus subcarinatus* | G | Log | 2 | 13475.61 | 0.87 | <0.001 | U | 0.2-0.7 | 0-3.0 |
| *Hyboscolex longiseta* | P | Log | 2 | 2021.35 | 0.41 | <0.001 | U | 0.3-1.5 | 0-3.0 |
| *Magelona dakini* | P | None | 2 | 18031.19 | 1.00 | >0.1 | n/a | n/a | 0-1.0 |
| Maldanidae | P | Log | 2 | 969.30 | 0.72 | <0.001 | SU | 0.2-1.0 | 0-3.0 |
| *Orbinia papillosa* | P | Square root | 1 | 3232.45 | 0.65 | <0.001 | D | 0.2-0.4 | 0-2.7 |
| *Owenia petersenae* | P | Square root | 2 | 5288.17 | 0.69 | <0.001 | U | 0.4-1.0 | 0-2.5 |
| *Scoloplos cylindrifer* | P | Log | 1 | 3756.58 | 0.61 | <0.001 | D | 0.2-0.5 | 0-3.0 |
| Terebellidae | P | Log | 2 | 2538.48 | 0.21 | <0.001 | U | 0.4-1.9 | 0.2-3.0 |

SM-4. Summary of generalised linear models predicting maximum density for 22 functional traits in response to sedimentation. Functional trait categories are: M = motility, F = feeding, H = habitat structure, S = sediment particle movement, BS = body size, BF = body form, L = living position in the sediment, BH = body hardness. Responses are: D = decline, SU = skewed unimodal, U = unimodal as represented in Figure 3. Optimum range indicates the stressor (% mud) range over which traits exhibit their highest abundances. Distribution range indicates the stressor range where at least one trait occurs.

| *Trait* | *Group* | *Predictor*  *transformation* | *Polynomial*  *order* | *Null*  *deviance* | *R^2^* | *p value* | *Response* | *Optimum* | *Distribution* |
| --- | --- | --- | --- | --- | --- | --- | --- | --- | --- |
| Freely motile | M | None | 2 | 13076.61 | 0.67 | <0.001 | D | 2.6-13.8 | <0.1-76.4 |
| Sedentary | M | None | 2 | 78658.32 | 0.74 | <0.001 | SU | 2.6-8.0 | 0-48.9 |
| Limited | M | Log | 1 | 17918.19 | 0.88 | <0.001 | D | 2.6-5.3 | 0-48.9 |
| Deposit | F | Log | 1 | 19859.91 | 0.70 | <0.001 | D | 2.6-7.8 | <0.1-76.4 |
| Suspension | F | Log | 2 | 73602.77 | 0.84 | <0.001 | D | 2.6-7.5 | <0.1-76.4 |
| Simple hole or pit | H | Log | 1 | 22573.28 | 0.87 | <0.001 | D | 2.6-5.0 | <0.1-48.9 |
| Permanent burrow | H | Log | 2 | 7129.61 | 0.54 | <0.001 | U | 2.6-22.4 | <0.1-76.4 |
| Trough | H | Log | 1 | 24427.08 | 0.93 | <0.001 | D | 2.6-4.5 | <0.1-76.4 |
| Tube | H | None | 2 | 67426.58 | 0.53 | <0.001 | U | 6.3-12.3 | 0.6-48.9 |
| Surface mixing | S | Log | 1 | 40559.09 | 0.83 | <0.001 | D | 2.6-5.0 | <0.1-76.4 |
| Deep-to-surface | S | Log | 2 | 10018.13 | 0.54 | <0.001 | D | 2.6-14.8 | <0.1-76.4 |
| Surface-to-deep | S | Log | 2 | 27535.11 | 0.30 | <0.001 | U | 3.3-16.5 | <0.1-76.4 |
| Small | BS | Square root | 1 | 56314.95 | 0.59 | <0.001 | D | 2.6-7.2 | <0.1-76.4 |
| Medium | BS | Log | 2 | 31972.81 | 0.75 | <0.001 | SU | 2.6-11.3 | <0.1-76.4 |
| Large | BS | Log | 2 | 12200.34 | 0.82 | <0.001 | SU | 2.6-11.7 | <0.1-76.4 |
| Round/globulose | BF | Log | 2 | 67561.89 | 0.88 | <0.001 | D | 2.6-7.5 | <0.1-76.4 |
| Worm-shaped | BF | Log | 2 | 30371.46 | 0.42 | <0.001 | SU | 2.6-15.4 | <0.1-76.4 |
| Top 2 cm | L | Log | 1 | 41805.74 | 0.94 | <0.001 | D | 2.6-4.8 | <0.1-76.4 |
| Deeper than 2 cm | L | None | 2 | 9402.70 | 0.63 | <0.001 | D | 2.6-14.5 | <0.1-76.4 |
| Attached | L | Log | 2 | 78782.23 | 0.94 | <0.001 | D | 2.6-5.7 | <0.1-48.9 |
| Soft-bodied | BH | Log | 2 | 32196.79 | 0.63 | <0.001 | SU | 2.6-13.9 | <0.1-76.4 |
| Calcified | BH | Square root | 1 | 45001.71 | 0.81 | <0.001 | D | 2.6-6.5 | <0.1-76.4 |

SM-5. Summary of generalised linear models predicting maximum density for 22 functional traits in response to nutrients. Functional trait categories are: M = motility, F = feeding, H = habitat structure, S = sediment particle movement, BS = body size, BF = body form, L = living position in the sediment, BH = body hardness. Responses are: D = decline, SU = skewed unimodal, U = unimodal as represented in Figure 3. Optimum range indicates the stressor (PC1 nutrients) range over which traits exhibit their highest abundances. Distribution range indicates the stressor range where at least one trait occurs.

| *Trait* | *Group* | *Predictor*  *transformation* | *Polynomial*  *order* | *Null*  *deviance* | *R^2^* | *p value* | *Response* | *Optimum* | *Distribution* |
| --- | --- | --- | --- | --- | --- | --- | --- | --- | --- |
| Freely motile | M | Log | 2 | 15306.53 | 0.46 | <0.001 | U | 0.6-4.2 | 0-10.01 |
| Sedentary | M | Square root | 2 | 66607.01 | 0.71 | <0.001 | U | 1.1-2.5 | 0-5.16 |
| Limited | M | Square root | 2 | 14648.37 | 0.89 | <0.001 | U | 0.8-2.6 | 0-5.16 |
| Deposit | F | Log | 2 | 17373.41 | 0.71 | <0.001 | U | 0.8-3.6 | 0-10.01 |
| Suspension | F | Log | 2 | 44850.80 | 0.86 | <0.001 | U | 0.9-2.4 | 0-10.01 |
| Simple hole or pit | H | Square root | 2 | 17310.22 | 0.86 | <0.001 | U | 0.8-2.5 | 0-5.16 |
| Permanent burrow | H | Log | 2 | 14898.21 | 0.40 | <0.001 | U | 0.9-5.5 | 0-10.01 |
| Trough | H | Square root | 2 | 15334.06 | 0.58 | <0.001 | U | 0.7-2.6 | 0-10.01 |
| Tube | H | None | 2 | 86517.70 | 0.61 | <0.001 | U | 1.8-2.6 | 0-4.47 |
| Surface mixing | S | Log | 2 | 31517.51 | 0.89 | <0.001 | U | 0.8-2.4 | 0-10.01 |
| Deep-to-surface | S | Log | 2 | 12088.71 | 0.26 | <0.001 | U | 0.7-4.9 | 0-10.01 |
| Surface-to-deep | S | Square root | 2 | 39388.55 | 0.35 | <0.001 | U | 1.5-3.6 | 0-10.01 |
| Small | BS | Log | 2 | 40493.89 | 0.66 | <0.001 | U | 0.9-3.0 | 0-10.01 |
| Medium | BS | Log | 2 | 32432.50 | 0.48 | <0.001 | U | 0.9-3.0 | 0-10.01 |
| Large | BS | Log | 2 | 8542.45 | 0.86 | <0.001 | U | 0.6-3.0 | 0-10.01 |
| Round/globulose | BF | Log | 2 | 44114.84 | 0.93 | <0.001 | U | 0.8-2.3 | 0-10.01 |
| Worm-shaped | BF | Square root | 2 | 43805.90 | 0.35 | <0.001 | U | 1.1-3.9 | 0-10.01 |
| Top 2 cm | L | Log | 2 | 29787.67 | 0.51 | <0.001 | U | 0.7-2.9 | 0-10.01 |
| Deeper than 2 cm | L | Log | 2 | 13912.40 | 0.35 | <0.001 | U | 0.8-5.5 | 0-10.01 |
| Attached | L | Log | 2 | 56471.94 | 0.83 | <0.001 | U | 0.9-2.0 | 0-5.16 |
| Soft-bodied | BH | Square root | 2 | 37481.18 | 0.59 | <0.001 | U | 1.1-3.4 | 0-10.01 |
| Calcified | BH | Square root | 2 | 34086.06 | 0.96 | <0.001 | U | 0.8-2.6 | 0-10.01 |

SM-6. Summary of generalised linear models predicting maximum density for 22 functional traits in response to metals. Functional trait categories are: M = motility, F = feeding, H = habitat structure, S = sediment particle movement, BS = body size, BF = body form, L = living position in the sediment, BH = body hardness. Responses are: D = decline, SU = skewed unimodal, U = unimodal as represented in Figure 3. Optimum range indicates the stressor (PC1 metals) range over which traits exhibit their highest abundances. Distribution range indicates the stressor range where at least one trait occurs.

| *Trait* | *Group* | *Predictor*  *transformation* | *Polynomial*  *order* | *Null*  *deviance* | *R^2^* | *p value* | *Response* | *Optimum* | *Distribution* |
| --- | --- | --- | --- | --- | --- | --- | --- | --- | --- |
| Freely motile | M | None | 2 | 12333.67 | 0.49 | <0.001 | D | 0.2-1.6 | 0-9.72 |
| Sedentary | M | Log | 2 | 85308.86 | 0.90 | <0.001 | SU | 0.2-0.6 | 0-4.15 |
| Limited | M | Log | 2 | 13108.70 | 0.70 | <0.001 | SU | 0.2-1.2 | 0-4.15 |
| Deposit | F | Log | 2 | 18074.66 | 0.41 | <0.001 | SU | 0.2-1.6 | 0-9.72 |
| Suspension | F | None | 2 | 67460.45 | 0.83 | <0.001 | D | 0.2-0.5 | 0-9.72 |
| Simple hole or pit | H | Log | 2 | 16616.26 | 0.71 | <0.001 | U | 0.2-1.2 | 0-4.15 |
| Permanent burrow | H | Square root | 2 | 8726.62 | 0.41 | <0.001 | D | 0.2-9.7 | 0-9.72 |
| Trough | H | Log | 2 | 21537.82 | 0.77 | <0.001 | U | 0.2-0.9 | 0-9.72 |
| Tube | H | Log | 2 | 103694.02 | 0.89 | <0.001 | U | 0.3-0.7 | 0-2.96 |
| Surface mixing | S | Log | 2 | 38924.51 | 0.86 | <0.001 | D | 0.2-0.7 | 0-9.72 |
| Deep-to-surface | S | Log | 1 | 9142.88 | 0.54 | <0.001 | D | 0.2-0.8 | 0-9.72 |
| Surface-to-deep | S | Log | 2 | 41540.58 | 0.56 | <0.001 | U | 0.2-1.0 | 0-9.72 |
| Small | BS | Log | 2 | 58456.80 | 0.72 | <0.001 | D | 0.2-0.6 | 0-9.72 |
| Medium | BS | Log | 2 | 28919.36 | 0.69 | <0.001 | U | 0.2-1.1 | 0-9.72 |
| Large | BS | Log | 2 | 8751.88 | 0.68 | <0.001 | D | 0.2-1.2 | 0-9.72 |
| Round/globulose | BF | Log | 2 | 66122.32 | 0.84 | <0.001 | D | 0.2-0.7 | 0-9.72 |
| Worm-shaped | BF | Log | 2 | 42323.85 | 0.53 | <0.001 | U | 0.2-1.2 | 0-9.72 |
| Top 2 cm | L | Log | 2 | 35550.70 | 0.67 | <0.001 | U | 0.2-1.2 | 0-9.72 |
| Deeper than 2 cm | L | Square root | 2 | 8716.45 | 0.52 | <0.001 | D | 0.2-1.1 | 0-9.72 |
| Attached | L | Square root | 1 | 72550.10 | 0.81 | <0.001 | D | 0.2-0.3 | 0-4.15 |
| Soft-bodied | BH | Log | 2 | 41038.27 | 0.74 | <0.001 | SU | 0.2-1.0 | 0-9.72 |
| Calcified | BH | Log | 2 | 48620.48 | 0.83 | <0.001 | SU | 0.2-0.8 | 0-9.72 |

SM-7. Plots of the relationship between individual taxa (as indicated) and percentage mud in the sediment. Each grey point is a single core, with 10 cores taken from each of 75 sites. Black points show the binned data. The generalised linear model for maximum abundance of the binned data is shown.


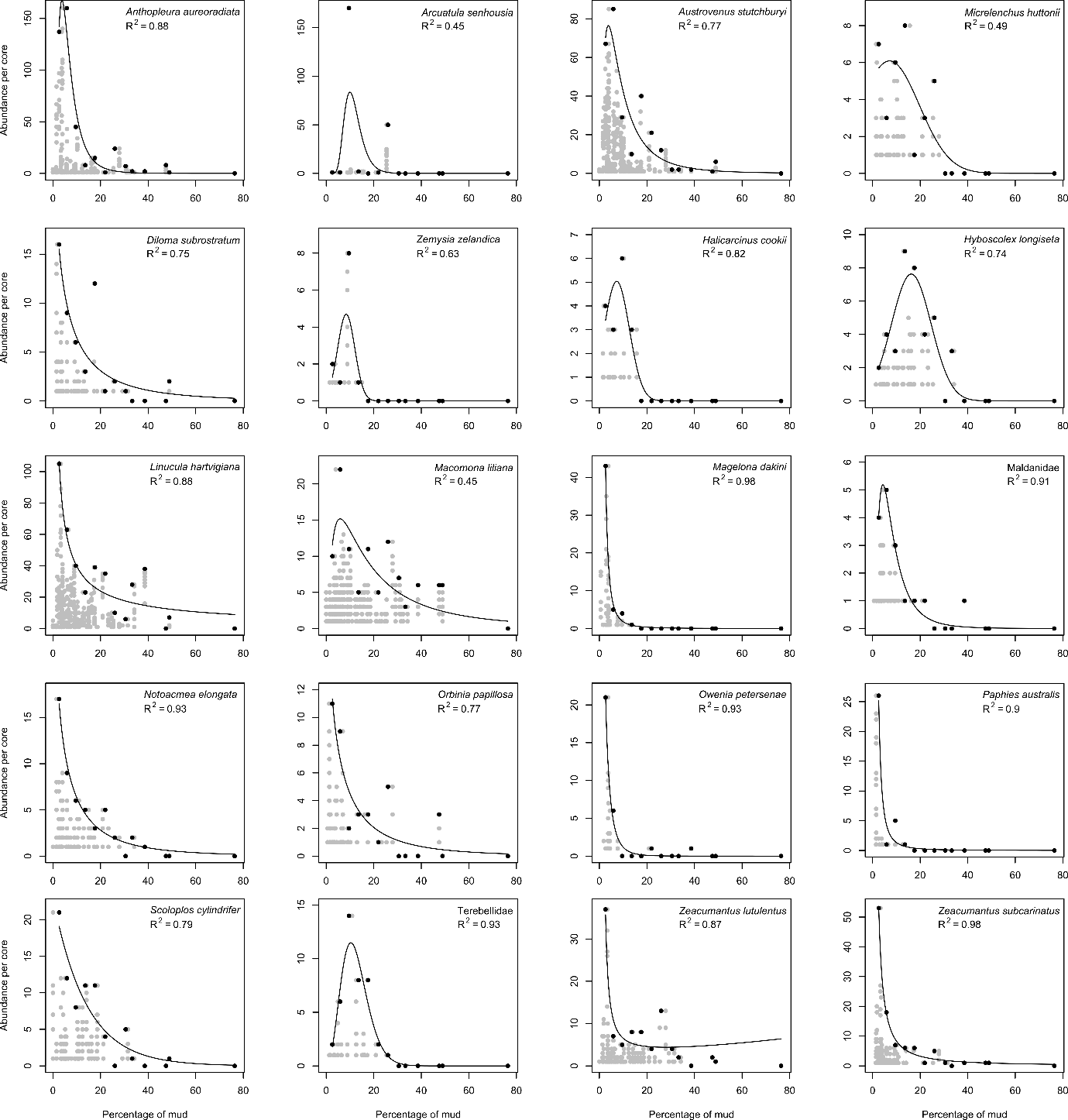


SM-8. Plots of the relationship between individual taxa (as indicated) and nutrients (PC1 nutrients) in the sediment. Each grey point is a single core, with 10 cores taken from each of 75 sites. Black points show the binned data. The generalised linear model for maximum abundance of the binned data is shown.


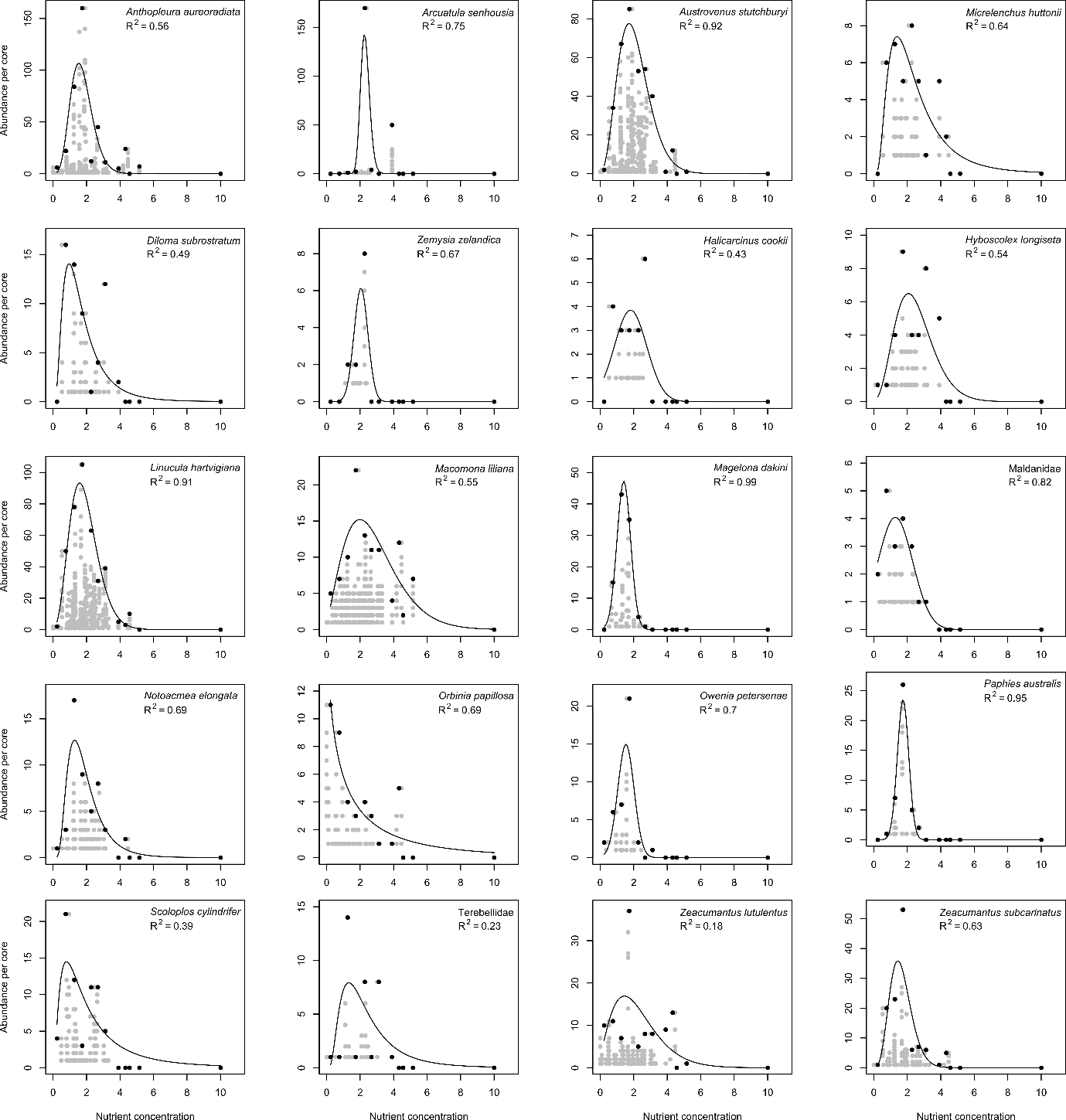


SM-9. Plots of the relationship between individual taxa (as indicated) and metals (PC1 metals). Each grey point is a single core, with 10 cores taken from each of 75 sites. Black points show the binned data. The generalised linear model for maximum abundance of the binned data is shown. The plot for *Magelona dakini* is not shown because it was not significant.


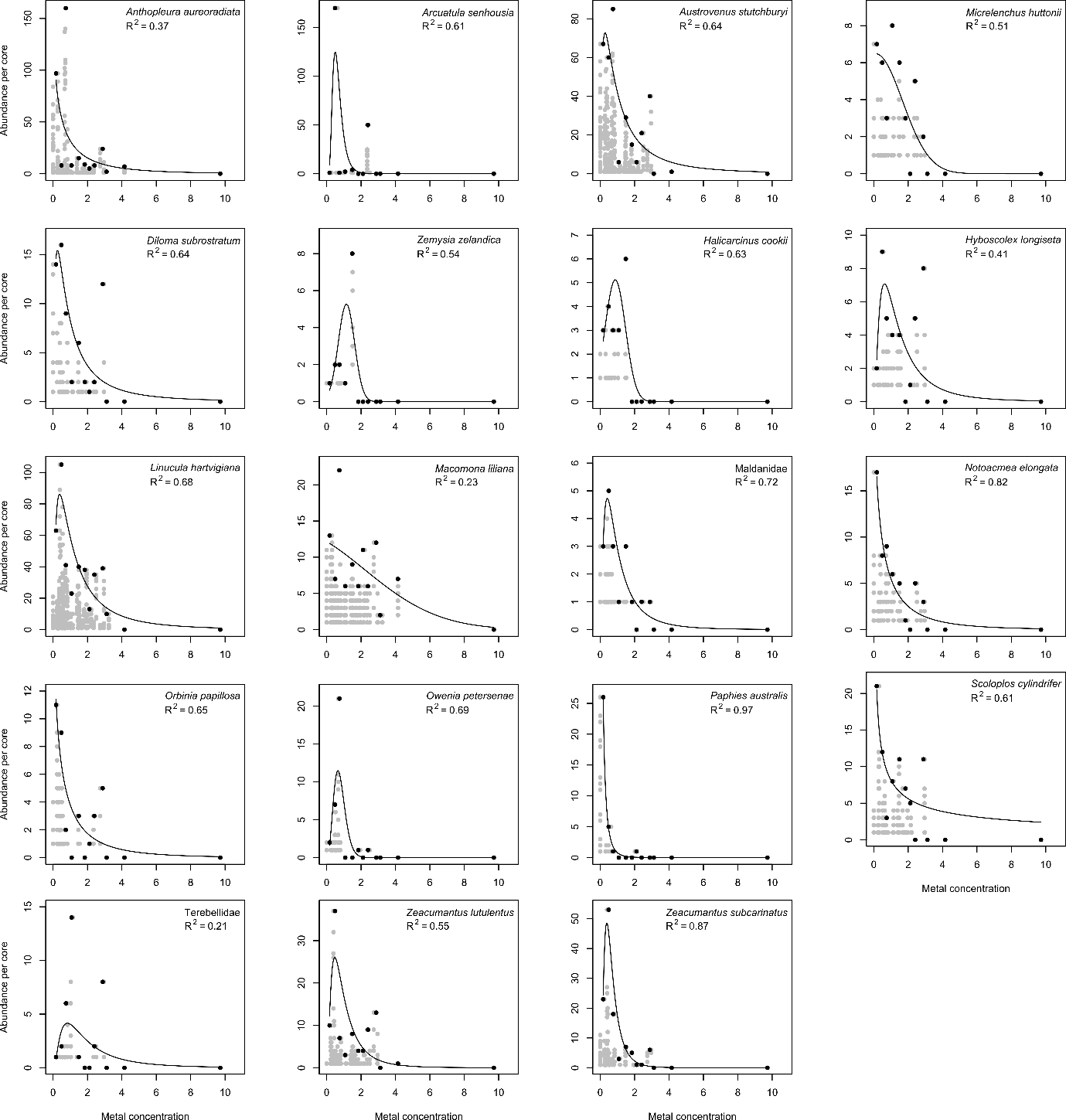


SM-10. Plots of the relationship between individual traits (as indicated) and percentage mud in the sediment. Each grey point is a single core, with 10 cores taken from each of 75 sites. Black points show the binned data. The generalised linear model for maximum abundance of the binned data is shown.


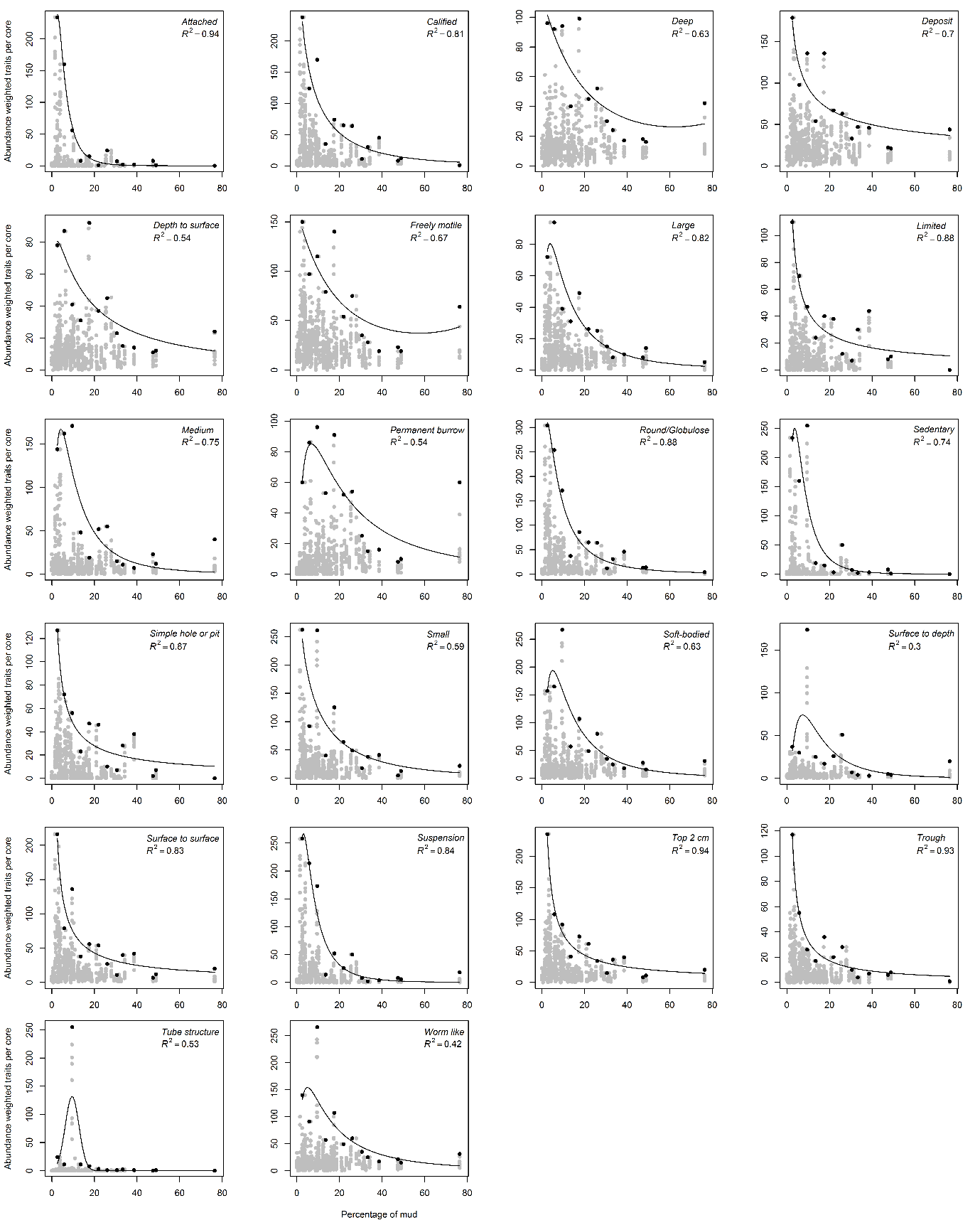


SM-11. Plots of the relationship between individual traits (as indicated) and nutrients (PC1 nutrients). Each grey point is a single core, with 10 cores taken from each of 75 sites. Black points show the binned data. The generalised linear model for maximum abundance of the binned data is shown.


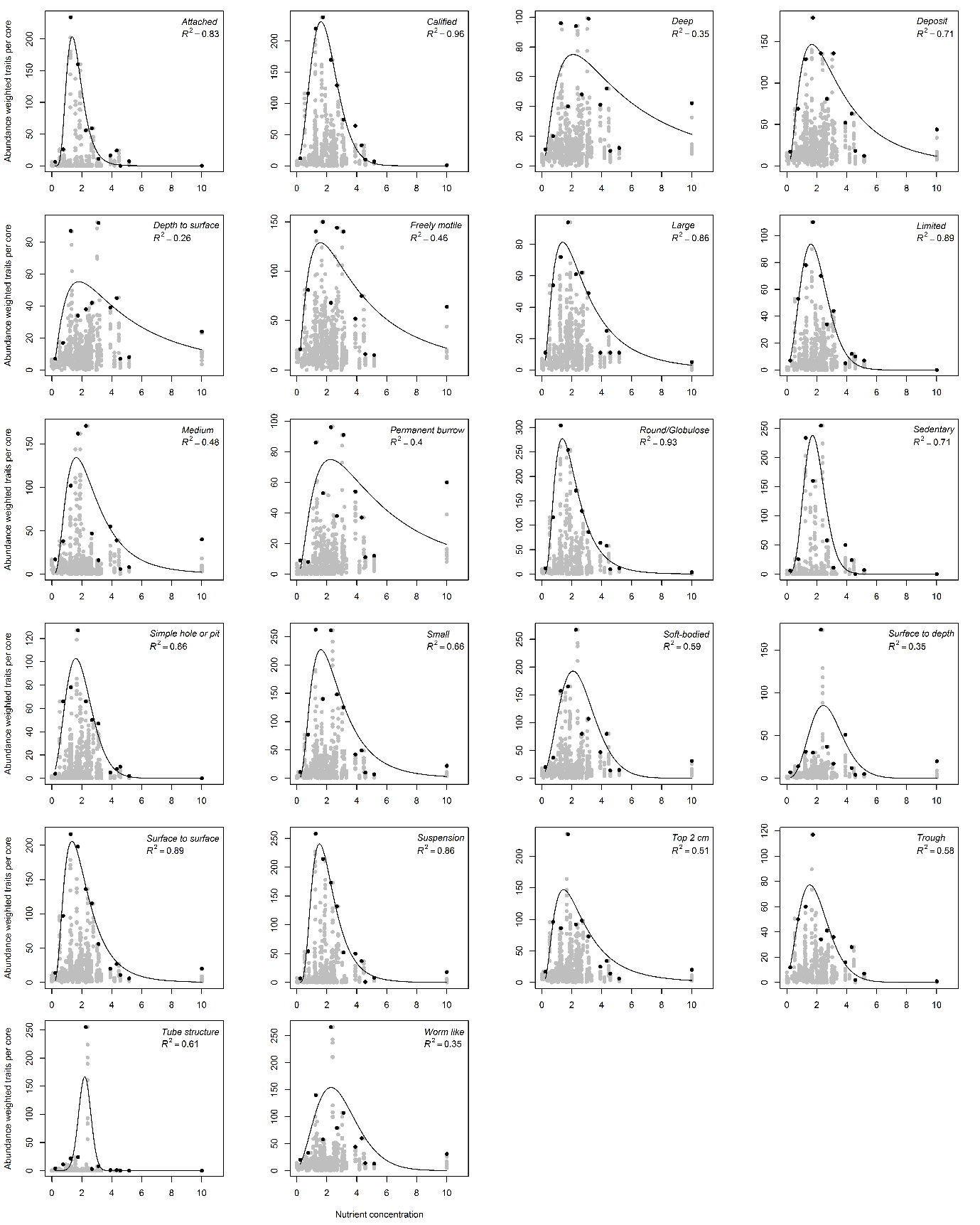


SM-12. Plots of the relationship between individual traits (as indicated) and metals (PC1 metals). Each grey point is a single core, with 10 cores taken from each of 75 sites. Black points show the binned data. The generalised linear model for maximum abundance of the binned data is shown.


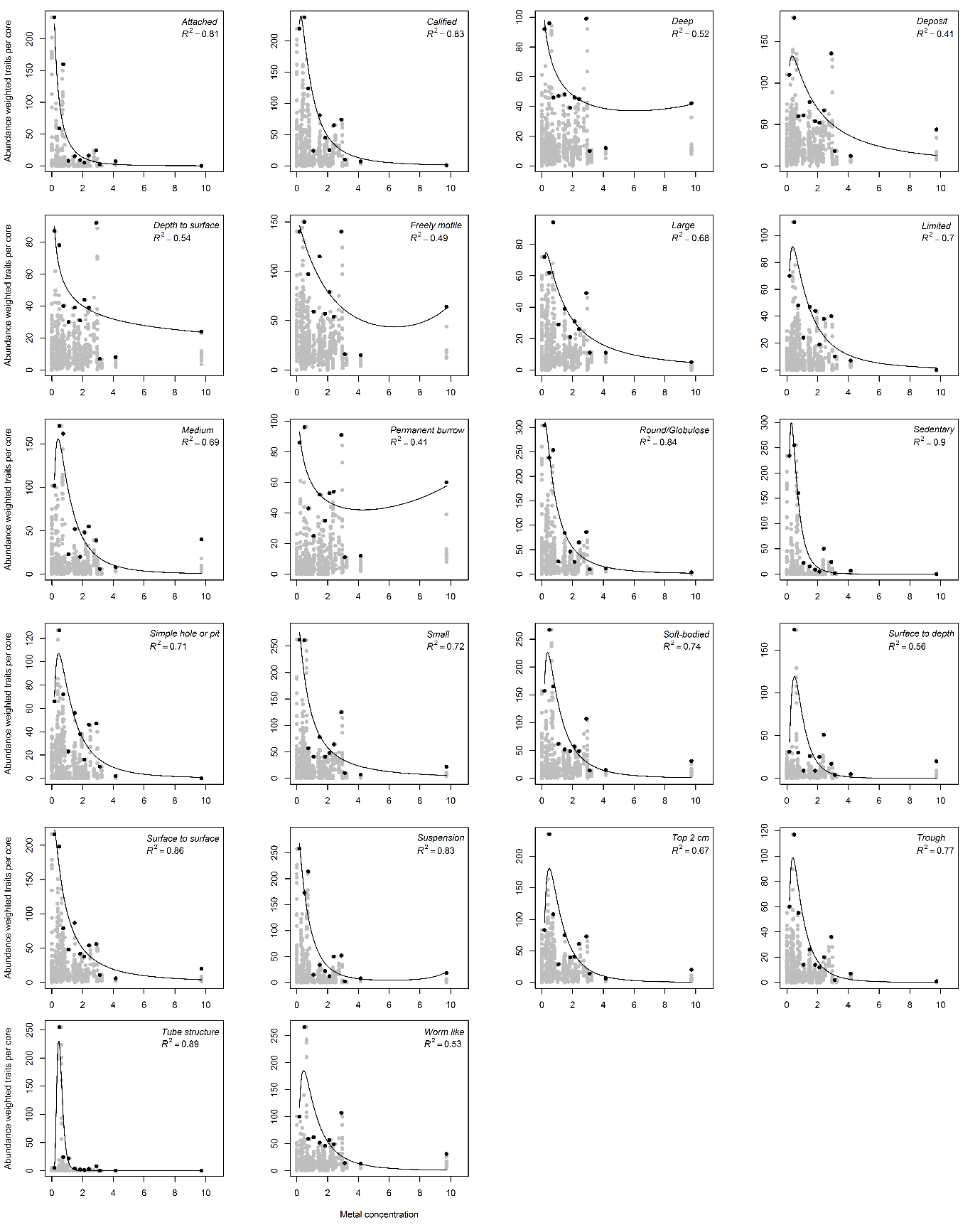


SM-13. Statistical models relating taxa abundance to variations in sediment grain size (percentage mud), nutrients (PC1 nutrients) and metals (PC1 metals). Bold indicates the presence of additive and multiplicative effects where antagonistic (Ant) interactions are defined by a significant negative estimate for an interaction term and both stressors acting in the same direction, synergistic (Syn) interactions are defined by a significant positive estimate and both stressors acting in the same direction, opposing synergistic effects (Op Syn) interactions are defined by a significant positive estimate and stressors acting in opposing directions and additive (Add) effects are defined by a significant estimate independent of other terms in the model. P-values are * < 0.05, ** < 0.01, *** < 0.001.

| *Taxa ^a^* | *Interaction* | *Parameter* | *Estimate* | *Std Error* | *p-value* |
| --- | --- | --- | --- | --- | --- |
| Anthozoans |  |  |  |  |  |
| *Anthopleura aureoradiata* |  | Sediments | -2.18 | 0.17 | *** |
| R^2^ = 0.41 |  | Nutrients | 1.76 | 0.11 | *** |
|  |  | Metals | -0.04 | 0.16 | 0.82 |
|  | **Syn** | **Sediments*Metals** | 1.01 | 0.33 | ** |
|  | **Ant** | **Nutrients*Metals** | -0.68 | 0.14 | *** |
| Bivalves |  |  |  |  |  |
| *Arcuatula senhousia* |  | Sediments | 0.34 | 0.14 | * |
| R^2^ = 0.33 |  | Nutrients | 0.80 | 0.09 | *** |
|  |  | Metals | -0.56 | 0.13 | *** |
|  | **Syn** | **Sediments*Nutrients** | 0.91 | 0.23 | *** |
|  | **Op Syn** | **Sediments*Metals** | 1.29 | 0.28 | *** |
|  | **Ant** | **Nutrients*Metals** | -0.59 | 0.22 | ** |
| *Austrovenus stutchburyi* | **Add** | **Sediments** | -1.96 | 0.13 | *** |
| R^2^ = 0.44 |  | Nutrients | 1.28 | 0.09 | *** |
|  |  | Metals | 0.00 | 0.12 | 0.99 |
|  | **Ant** | **Nutrients*Metals** | -0.40 | 0.24 | 0.09 |
| *Linucula hartvigiana* |  | Sediments | -1.21 | 0.14 | *** |
| R^2^ = 0.17 |  | Nutrients | 0.47 | 0.10 | *** |
|  |  | Metals | 0.50 | 0.14 | *** |
|  | **Ant** | **Sediments*Nutrients** | -0.63 | 0.26 | * |
|  | **Syn** | **Nutrients*Metals** | 1.28 | 0.28 | *** |
| *Macomona liliana* |  | Sediments | 0.05 | 0.08 | 0.55 |
| R^2^ = 0.14 |  | Nutrients | 0.43 | 0.05 | *** |
|  |  | Metals | -0.51 | 0.07 | *** |
|  | **Syn** | **Sediments*Nutrients** | 0.34 | 0.07 | *** |
|  | **Ant** | **Sediments*Metals** | -0.26 | 0.06 | *** |
| *Paphies australis* |  | Sediments | -0.31 | 0.08 | *** |
| R^2^ = 0.32 |  | Nutrients | 0.38 | 0.09 | *** |
|  |  | Metals | -0.38 | 0.10 | *** |
|  | **Syn** | **Sediments*Metals** | 0.59 | 0.08 | *** |
| Gastropods |  |  |  |  |  |
| *Notoacmea elongata* |  | Sediments | -0.36 | 0.10 | *** |
| R^2^ = 0.14 |  | Nutrients | 0.24 | 0.07 | *** |
|  |  | Metals | -0.05 | 0.10 | 0.60 |
|  | **Ant** | **Sediments*Nutrients** | -0.45 | 0.09 | *** |
|  | **Syn** | **Sediments*Metals** | 0.28 | 0.08 | *** |
| *Zeacumantus subcarinatus* |  | Sediments | -1.00 | 0.13 | *** |
| R^2^ = 0.21 |  | Nutrients | 0.30 | 0.09 | *** |
|  |  | Metals | 0.26 | 0.13 | * |
|  | **Op syn** | **Sediment*Metals** | 0.43 | 0.27 | 0.11 |
|  | **Syn** | **Nutrients*Metals** | 0.37 | 0.23 | 0.11 |
| Polychaetes |  |  |  |  |  |
| *Hyboscolex longiseta* |  | Sediments | 0.37 | 0.11 | *** |
| R^2^ = 0.15 |  | Nutrients | -0.10 | 0.08 | 0.20 |
|  |  | Metals | -0.14 | 0.10 | 0.17 |
|  | **Ant** | **Sediments*Metals** | -0.29 | 0.15 | * |
|  | **Syn** | **Nutrients*Metals** | 0.69 | 0.18 | *** |
| *Magelona dakini* |  | Sediments | -0.79 | 0.12 | *** |
| R^2^ = 0.27 |  | Nutrients | 0.23 | 0.10 | * |
|  |  | Metals | 0.14 | 0.13 | 0.29 |
|  | **Op Syn** | **Sediments*Nutrients** | 0.37 | 0.20 | 0.07 |
|  | **Syn** | **Sediments*Metals** | 0.75 | 0.26 | ** |
|  | **Syn** | **Nutrients*Metals** | 0.94 | 0.27 | *** |
| *Orbinia papillosa* |  | Nutrients | -0.25 | 0.06 | *** |
| R^2^ = 0.45 |  | Metals | -0.10 | 0.08 | 0.19 |
|  | **Ant** | **Nutrients*Metals** | -0.70 | 0.07 | *** |

^a^ R^2^ values < 0.1 indicated that the models for *Zemysia zelandica, Halicarcinus cookii, Diloma subrostratum, Micrelenchus huttonii, Zeacumantus lutulentus,* Maldanidae, *Owenia petersenae, Scoloplos cylindrifer* and Terebellidae did not fit well so these taxa are not included in this table.

SM-14. Statistical models relating trait abundance to variations in sediment grain size (percentage mud), nutrients (PC1 nutrients) and metals (PC1 metals). Bold indicates the presence of additive and multiplicative effects where antagonistic (Ant) interactions are defined by a significant negative estimate for an interaction term and both stressors acting in the same direction, synergistic (Syn) interactions are defined by a significant positive estimate and both stressors acting in the same direction, opposing synergistic effects (Op Syn) interactions are defined by a significant positive estimate and stressors acting in opposing directions and additive (Add) effects are defined by a significant estimate independent of other terms in the model. P-values are * < 0.05, ** < 0.01, *** < 0.001.

| *Traits* | *Interaction* | *Parameter* | *Estimate* | *Std Error* | *p-value* |
| --- | --- | --- | --- | --- | --- |
| Motility |  |  |  |  |  |
| Freely motile |  | Sediments | -0.68 | 0.07 | *** |
| R^2^ = 0.25 |  | Nutrients | 0.58 | 0.06 | *** |
|  |  | Metals | -0.03 | 0.07 | 0.72 |
|  | **Op Syn** | **Sediments*Nutrients** | 0.41 | 0.08 | *** |
|  | **Ant** | **Nutrients*Metals** | -0.49 | 0.15 | ** |
| Sedentary |  | Sediments | -2.16 | 0.19 | *** |
| R^2^ = 0.45 |  | Nutrients | 2.01 | 0.13 | *** |
|  |  | Metals | -0.45 | 0.18 | * |
|  | **Ant** | **Sediments*Nutrients** | -0.63 | 0.35 | 0.07 |
|  | **Syn** | **Sediments*Metals** | 1.61 | 0.38 | *** |
|  | **Ant** | **Nutrients*Metals** | -0.87 | 0.37 | * |
| Limited | **Add** | **Sediments** | -0.82 | 0.13 | *** |
| R^2^ = 0.17 |  | Nutrients | 0.66 | 0.09 | *** |
|  |  | Metals | 0.06 | 0.12 | 0.59 |
|  | **Syn** | **Nutrients*Metals** | 0.77 | 0.23 | *** |
| Feeding |  |  |  |  |  |
| Deposit |  | Sediments | -0.49 | 0.08 | *** |
| R^2^ = 0.14 |  | Nutrients | 0.55 | 0.06 | *** |
|  |  | Metals | -0.05 | 0.08 | 0.54 |
|  | **Op Syn** | **Sediments*Nutrients** | 0.37 | 0.09 | *** |
| Suspension |  | Sediments | -1.97 | 0.14 | *** |
| R^2^ = 0.48 |  | Nutrients | 1.71 | 0.10 | *** |
|  |  | Metals | -0.24 | 0.14 | 0.08 |
|  | **Syn** | **Sediments*Metals** | 0.97 | 0.28 | *** |
| Habitat structure |  |  |  |  |  |
| Simple hole or pit | **Add** | **Sediments** | -1.39 | 0.14 | *** |
| R^2^ = 0.25 |  | Nutrients | 0.80 | 0.10 | *** |
|  |  | Metals | 0.25 | 0.14 | 0.07 |
|  | **Op Syn** | **Nutrients*Metals** | 0.42 | 0.12 | *** |
| Permanent burrow |  | Sediments | 0.31 | 0.13 | * |
| R^2^ = 0.19 |  | Nutrients | 0.27 | 0.09 | ** |
|  |  | Metals | 0.01 | 0.12 | 0.93 |
|  | **Syn** | **Sediments*Nutrients** | 1.13 | 0.23 | *** |
|  | **Ant** | **Sediment*Metals** | -0.76 | 0.26 | ** |
| Trough | **Add** | **Sediments** | -1.17 | 0.07 | *** |
| R^2^ = 0.27 | **Add** | **Nutrients** | 0.79 | 0.07 | *** |
| Tube | **Add** | **Sediments** | -0.44 | 0.17 | * |
| R^2^ = 0.33 |  | Nutrients | 1.23 | 0.19 | *** |
|  |  | Metals | -1.23 | 0.22 | *** |
|  | **Ant** | **Nutrients*Metals** | -1.89 | 0.52 | *** |
| Sediment particle movement | |  |  |  |  |
| Surface mixing |  | Sediment | -1.03 | 0.11 | *** |
| R^2^ = 0.32 |  | Nutrients | 0.68 | 0.08 | *** |
|  |  | Metals | -0.03 | 0.11 | 0.81 |
|  | **Syn** | **Sediments*Metals** | 0.30 | 0.08 | *** |
| Deep-to-surface |  | Sediment | -0.48 | 0.08 | *** |
| R^2^ = 0.17 |  | Nutrients | 0.64 | 0.07 | *** |
|  |  | Metals | -0.09 | 0.09 | 0.33 |
|  | **Op Syn** | **Sediments*Nutrients** | 0.66 | 0.13 | *** |
|  | **Ant** | **Sediments*Metals** | -0.64 | 0.17 | *** |
|  | **Ant** | **Nutrients*Metals** | -0.27 | 0.18 | 0.14 |
| Surface-to-deep |  | Sediments | -0.59 | 0.10 | *** |
| R^2^ = 0.34 |  | Nutrients | 0.99 | 0.08 | *** |
|  |  | Metals | -0.47 | 0.11 | *** |
|  | **Op Syn** | **Sediments*Nutrients** | 0.23 | 0.11 | * |
|  | **Ant** | **Nutrients*Metals** | -0.66 | 0.16 | *** |
| Body size |  |  |  |  |  |
| Small | **Add** | **Sediment** | -0.88 | 0.13 | *** |
| R^2^ = 0.21 | **Add** | **Nutrients** | 0.92 | 0.09 | *** |
|  | **Add** | **Metals** | -0.22 | 0.13 | 0.09 |
| Medium |  | Sediment | -0.92 | 0.11 | *** |
| R^2^ = 0.21 |  | Nutrients | 0.69 | 0.09 | *** |
|  |  | Metals | 0.00 | 0.12 | 0.97 |
|  | **Op Syn** | Sediments*Nutrients | 0.58 | 0.16 | *** |
|  | **Syn** | Sediments*Metals | 0.35 | 0.16 | * |
|  | **Ant** | Nutrients*Metals | -0.47 | 0.19 | ** |
| Large | **Add** | **Sediments** | -0.98 | 0.08 | *** |
| R^2^ = 0.33 |  | Nutrients | 0.77 | 0.06 | *** |
|  |  | Metals | -0.04 | 0.08 | 0.62 |
|  | **Ant** | **Nutrients*Metals** | -0.50 | 0.07 | *** |
| Body form |  |  |  |  |  |
| Round/globulose |  | Sediments | -1.42 | 0.12 | *** |
| R^2^ = 0.36 |  | Nutrients | 1.12 | 0.08 | *** |
|  |  | Metals | -0.03 | 0.12 | 0.77 |
|  | **Syn** | **Sediments*Metals** | 0.58 | 0.24 | * |
| Worm-shaped |  | Sediments | -0.32 | 0.11 | ** |
| R^2^ = 0.14 |  | Nutrients | 0.58 | 0.09 | *** |
|  |  | Metals | -0.27 | 0.12 | * |
|  | **Op Syn** | **Sediments*Nutrients** | 0.53 | 0.16 | *** |
|  | **Ant** | **Sediments*Metals** | -0.79 | 0.22 | *** |
|  | **Ant** | **Nutrients*Metals** | -0.71 | 0.17 | *** |
| Living position in the sediment | | |  |  |  |
| Top 2 cm |  | Sediments | -1.08 | 0.10 | *** |
| R^2^ = 0.33 |  | Nutrients | 0.70 | 0.07 | *** |
|  |  | Metals | 0.05 | 0.09 | 0.59 |
|  | **Op Syn** | **Sediments*Metals** | 0.22 | 0.07 | ** |
| Deeper than 2 cm |  | Sediments | -0.05 | 0.10 | 0.58 |
| R^2^ = 0.14 |  | Nutrients | 0.50 | 0.07 | *** |
|  |  | Metals | -0.29 | 0.09 | ** |
|  | **Op Syn** | **Sediment*Nutrients** | 0.80 | 0.18 | *** |
|  | **Ant** | **Sediment*Metals** | -0.64 | 0.19 | *** |
|  | **Ant** | **Nutrients*Metals** | -0.40 | 0.19 | * |
| Attached |  | Sediments | -2.26 | 0.19 | *** |
| R^2^ = 0.45 |  | Nutrients | 1.77 | 0.13 | *** |
|  |  | Metals | -0.15 | 0.18 | 0.41 |
|  | **Ant** | **Sediments*Nutrients** | -0.33 | 0.30 | 0.29 |
|  | **Syn** | **Sediments*Metals** | 1.34 | 0.38 | *** |
|  | **Ant** | **Nutrients*Metals** | -0.41 | 0.31 | 0.19 |
| Body hardness |  |  |  |  |  |
| Soft-bodied |  | Sediments | -0.72 | 0.10 | *** |
| R^2^ = 0.21 |  | Nutrients | 0.84 | 0.08 | *** |
|  |  | Metals | -0.20 | 0.11 | 0.07 |
|  | **Op Syn** | **Sediments*Nutrients** | 0.64 | 0.16 | *** |
|  | **Ant** | **Sediments*Metals** | -0.34 | 0.21 | 0.11 |
|  | **Ant** | **Nutrients*Metals** | -0.52 | 0.22 | * |
| Calcified |  | Sediments | -1.31 | 0.13 | *** |
| R^2^ = 0.30 |  | Nutrients | 1.00 | 0.09 | *** |
|  |  | Metals | -0.03 | 0.12 | 0.82 |
|  | **Syn** | **Sediments*Metals** | 0.43 | 0.25 | 0.09 |
|  | **Op Syn** | **Nutrients*Metals** | 0.41 | 0.24 | 0.09 |
